# Niacin ameliorates Charcot-Marie-Tooth 4B1 neuropathy without interfering with nerve regeneration

**DOI:** 10.1093/braincomms/fcaf039

**Published:** 2025-01-31

**Authors:** Silvia Cipriani, Emanuela Porrello, Matteo Cerea, Andrea Gazzaniga, Roberta Di Guardo, Amanda Heslegrave, Serena Valenzano, Ubaldo Del Carro, Phu Duong, John Svaren, Stefano Carlo Previtali, Alessandra Bolino

**Affiliations:** IRCCS Ospedale San Raffaele, Human Inherited Neuropathies Unit, Milan 20132, Italy; IRCCS Ospedale San Raffaele, Neuromuscular Repair Unit, Milan 20132, Italy; Department of Pharmaceutical Sciences, Università degli Studi di Milano, Milan 20133, Italy; Department of Pharmaceutical Sciences, Università degli Studi di Milano, Milan 20133, Italy; IRCCS Ospedale San Raffaele, Human Inherited Neuropathies Unit, Milan 20132, Italy; UKDRI Fluid Biomarker Laboratory, London WC1N3BG, UK; Department of Neurology, IRCCS Ospedale San Raffaele, Milan 20132, Italy; Department of Neurology, IRCCS Ospedale San Raffaele, Milan 20132, Italy; Waisman Center and Department of Comparative Biosciences, School of Veterinary Medicine, University of Wisconsin-Madison, Madison, WI 53705, USA; Waisman Center and Department of Comparative Biosciences, School of Veterinary Medicine, University of Wisconsin-Madison, Madison, WI 53705, USA; IRCCS Ospedale San Raffaele, Neuromuscular Repair Unit, Milan 20132, Italy; Department of Neurology, IRCCS Ospedale San Raffaele, Milan 20132, Italy; Vita-Salute San Raffaele University, Milan 20132, Italy; IRCCS Ospedale San Raffaele, Human Inherited Neuropathies Unit, Milan 20132, Italy; Vita-Salute San Raffaele University, Milan 20132, Italy

**Keywords:** Charcot-Marie-Tooth, neuropathy, myelin, nicotinic acid, nerve regeneration

## Abstract

Charcot-Marie-Tooth (CMT) neuropathies represent a broad and very heterogeneous group of disorders for which no therapies are yet available. Due to the huge genetic heterogeneity, therapeutical approaches that can benefit several forms independently of the unique pathogenetic mechanism have been sought. Niacin, nicotinic acid, is a vitamin used for many decades as anti-dyslipidaemic and anti-cholesterol drug product under the commercial name of Niaspan^®^, the extended-release formulation of niacin. Of note, niacin can have other effects depending on the dose, formulation and physiology and it has been used to reduce inflammation, to promote angiogenesis and to protect neurons, muscle and axons by boosting nicotinamide adenine dinucleotide (NAD^+^) levels. Niacin also activates TNF-alpha convertase enzyme (TACE) secretase, which negatively regulates Neuregulin type I-mediated signalling in the peripheral nervous system and myelination. We previously postulated that niacin-mediated TACE activation can be effective in reducing aberrant excessive myelin associated with different CMT forms. Here, we explored efficacy of this strategy by performing a long-term preclinical trial and we provided evidence that a novel niacin-based long-lasting formulation ameliorates neurophysiology and reduces fibre degeneration in a model of Charcot-Marie-Tooth type 4B1 (CMT4B1) neuropathy, characterized by aberrant myelin. We also sought to determine whether this strategy might interfere with nerve regeneration, which is dependent on Neuregulin type I signalling. Surprisingly, we found that the *Mtmr2* knockout mice, a model of CMT4B1, have a defect in nerve regeneration and that niacin-based treatment is not detrimental to nerve regeneration.

## Teaser

Cipriani *et al*. report that a novel long-lasting formulation of niacin (vitamin B3) ameliorates neurophysiology and reduces fibres degeneration in a mouse model of Charcot-Marie-Tooth (CMT) 4B1, a severe demyelinating neuropathy with aberrant myelin. These findings are clinically relevant as fibre degeneration and loss correlate with clinical disability in patients.

## Graphical Abstract

**graphical abstract:**
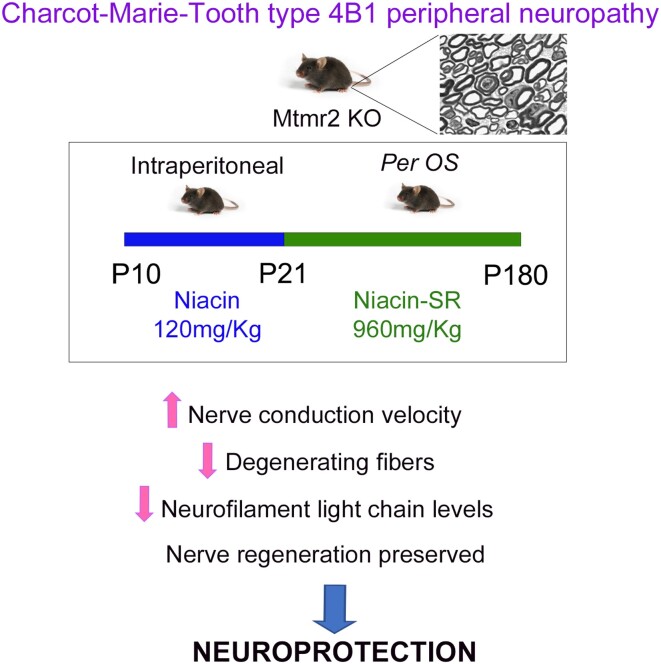

## Introduction

Charcot-Marie-Tooth (CMT) neuropathies represent a broad and heterogeneous group of disorders generally characterized by progressive muscular atrophy and weakness mainly occurring at distal extremities of the lower and upper limbs.^1-3^ On the basis of clinical and neurophysiological criteria, CMTs are distinguished in demyelinating, axonal and intermediate types.^4^ Among the demyelinating group, Charcot-Marie-Tooth type 4B1 (CMT4B1) represents an autosomal recessive neuropathy characterized by childhood onset and myelin outfoldings, a form of redundant and aberrant myelin.^5,6^ In patients with CMT4B1, the neuropathy rapidly progresses with loss of myelinated fibres, thus impacting nerve and muscle function and leading to severe clinical disability.

We first demonstrated that CMT4B1 is caused by loss-of-function mutations in the *MTMR2* gene (Myotubularin-related 2), which encodes a ubiquitously expressed phospholipid phosphatase acting on PtdIns3P and PtdIns(3,5)*P*_2_ phosphoinositides.^7^ We previously reported that loss of MTMR2 in Schwann cells is both sufficient and necessary to cause myelin outfoldings and aberrant myelin.^8,9^ However, due to the early onset of the neuropathy with fast progression, a cell autonomous role of MTMR2 in neurons/axons cannot be excluded.

The Myotubularin-related protein (MTMR) family consists of 14 members, including eight catalytically active and six catalytically inactive proteins.^10,11^ Heterodimers of active and inactive members possess higher enzymatic activity and are thought to be specifically recruited at sub-cellular membranes. MTMR2 interacts with either MTMR5 or MTMR13, both catalytically inactive members. Consistent with this, mutations in *MTMR13*, also known as SBF2, cause CMT4B2, a very similar phenotype as compared with CMT4B1, with myelin outfoldings and demyelination, although relatively less severe.^12,13^ Finally, mutations in *MTMR5*, also known as SBF1, cause CMT4B3, which can be associated with a complex central nervous system phenotype and, more rarely, with demyelination and myelin outfoldings.^5^

Axonal Neuregulin 1 (NRG1) type III is one of the main factors influencing Schwann cell development in the peripheral nervous system (PNS).^14-16^ At the onset of myelination, NRG1 dictates the fate of Schwann cells to become myelinating or non-myelinating and the amount of myelin that has to be produced, which is a function of axonal diameter. In the axon, NRG1 activity is modulated by secretase cleavage, namely BACE (Beta-secretase 1) and TNF-alpha convertase enzyme (TACE), which activates and inactivates the molecule, respectively.^17^ Interestingly, niacin, nicotinic acid or vitamin B3, have been found to be a TACE activator and thus a putative negative regulator of NRG1 type III in the PNS.^18^ The extended-release formulation of niacin, Niaspan^®^ (Rebel Distributors Corp.), is a FDA (Food and Drug Administration)-approved medicinal product used for many years to treat dyslipidaemia and atherosclerosis.^19^

We recently suggested that niacin treatment, by enhancing TACE and likely by reducing NRG1 type III-mediated signalling in Schwann cells, ameliorates CMT neuropathies associated with excessive aberrant myelin.^18^ NRG1 type III is crucial for all steps of nerve development and, importantly, it is also required for remyelination after injury, similarly to NRG1 type I, another NRG1 isoform mainly expressed by repair Schwann cells.^20-22^ Thus, in a perspective of a clinical translation of this strategy, we explored whether downregulation of NRG1 signalling via niacin-mediated TACE activation might be detrimental to nerve regeneration, which is dependent on NRG1. Then, we performed a long-term preclinical trial in the *Mtmr2* KO mouse, a model of CMT4B1, using an extended-release formulation of niacin administered *per os*, thus mimicking Niaspan, the FDA-approved niacin-based drug. Our data suggest that niacin treatment partially ameliorates the CMT4B1 neuropathy without interfering with nerve regeneration, which we found to be altered in *Mtmr2* KO mice.

## Materials and methods

### Ethics statement

Mice used in this study were maintained in a pathogen-free facility in the San Raffaele Hospital (Milan, Italy) according to the National Health Institute guidelines. All experiments were carried out following Italian national regulations and protocols previously accepted by local Institutional Animal Care and Use Committees.

### Mouse model and genotyping

*Mtmr2* KO mice were generated on a 129/SvPas background as previously described.^8^ DNA was isolated from mouse tails using Direct PCR lysis reagent (cat#102-T, Viagen, Biotech) and Proteinase K from Tritirachium album (P2308-100 mg, SIGMA, Merck Group, USA). PCR analysis was then performed using GoTaq G2 Flexi DNA Polymerase kit (M7806, PROMEGA, USA). Animals were randomly included into experimental groups according to genotyping, age and sex. No animals had to be excluded because of illness. Animal experiments (morphological analyses) were performed in a blinded fashion towards the investigator.

### Sciatic nerve crush

To perform nerve crush experiments, males were always used for both wild type (WT) and *Mtmr2* KO groups to minimize variability. 2-2-2-tribromoethanol (T48402, SIGMA, Merck Group, USA) was used as anaesthetic (0.02 mL/g of body weight) and administered by intraperitoneal (i.p.) injection prior to surgical intervention.

Mice were finely incised between the ‘gluteus’ and the ‘biceps femoris’ muscles to expose the sciatic nerve. The mechanical and thermic injury were obtained by stapling the nerve at proximal sites using pre-cooled forceps. To facilitate the identification of the injured area, the crush site was marked with carbon powder (SIGMA, Merck Group, USA). Finally, Monofilament Nylon Dermalon 4.0 (COVIDIEN) was used to suture the incision and mice monitored until their complete recovery from anaesthesia.

### Niacin *in vivo* treatment

Pure niacin (nicotinic acid, cell culture grade, SIGMA, Merck Group, USA) was dissolved in NaCl 0.9% (SALF) and a 120 mg/kg dose was daily administered to mice by intraperitoneal injection. Animal’s body weight and clinical signs were closely monitored every day.

Good-practice dose-volumes guidelines (International Consortium for Innovation and Quality in Pharmaceutical Development-IQ 3Rs Leadership Group) were applied for all treatments.

### Niacin-SR formulation

Niacin was encapsulated in prolonged release granules by applying an ethylcellulose-based film coating. Uncoated granules were prepared by high-shear mixer (Rotolab, Zanchetta, Italy) operating at 600 rpm using 150 g of PVP (polyvinylpyrrolidone) 5% w/w solution (water:ethanol 1:1) on 500 g of niacin (Ph.Eur grade, Acros Organics, Thermo Fisher, USA). Granules were dried in static oven (VT6060P, Heraeus, DE) at 40°C for 12 h. Coating procedure was performed on 125–355 µm granules in a fluid bed apparatus (GDPC 1.1, Glatt, DE) applying ethylcellulose aqueous dispersion (Surelease^®^, Colorcon, UK) supplemented with 5% w/w (based on solids) low-viscosity hypromellose (Methocel E5 premium LV, Colorcon, UK) at the following conditions: batch size 300 g, product temperature 39–40°C, inlet air temperature 49–54°C, fluidizing air volume 50–64 m^3^/h, nebulization pressure 2.0 bar, spray rate 4.5 g/min. A final curing phase at 60°C for 24 h in static oven was applied to stabilize the control release performance. Coated granules were sieved for removing those having size bigger than 500 µm. Stability of the new compound (Niacin-SR) was examined and confirmed at 25°C and 65% relative humidity for 6 months. *In vitro* release kinetics of Niacin-SR and of Niaspan^®^ 750 mg tablets were evaluated applying conditions of reported in the USP pharmacopoeia monograph for Niacin Extended-Release Tablets, Test 4. 900 mL water at 37°C were used as the medium in a paddle dissolution apparatus rotating at 100 rpm (USP type 2 apparatus, mod. AT7 Smart, Sotax, CH) and collecting samples at 1, 3, 6, 9 and 24 h. Niacin content was assayed on coated granules by crushing 5 g sample in a mortar grinder (RM 200, Retsch, DE) operating for 5 min and dissolving 100 mg of resulting powder in 100 mL methanol and water (82:18). After 5 min sonication (mod. 2800, Branson Ultrasonics, USA) solution was filtered on 0.45 µm filter and transferred in 1 mL glass vials for HPLC (High-performance liquid chromatography) analysis. High-performance liquid chromatographic method was applied for quantification of niacin in drug release studies and in coated granules using HPLC (Binary pump 1525, Waters, USA) equipped with an autosampler set a 4°C (Autosampler 717plus, Waters, USA) injecting a volume of 10 µL and using an UV-detector (Dual Lambda Absorbance Detector 2487, Waters, USA) set at 260 nm and using a column 4.6 mm × 15 mm; 5 μm packing L8 (Supelcosil, Supelco, USA). Mobile phase was methanol and water (82:18), adjusted to pH 3.15 ± 0.05 with acetic acid, delivered at 1.3 ml/min. Data were collected and processed using Breeze Software for HPLC (Waters, USA).

### Pharmacokinetics studies

Pure niacin or Niacin-SR was administered by intraperitoneal injection or gavage at different doses to WT 129sv/Pas mice every day from P30 to P37. On the last day, sciatic nerves were collected at fixed time-points after last administration, promptly snap-frozen in liquid nitrogen and pools of nerves from three animals per time-point were prepared to have enough material for further analyses. Pharmacokinetics studies were then performed in collaboration with Eurofins, ADME BIOANALYSES (France).

Acetonitrile and water were used to prepare a calibration standard curve ranging from 100 to 200.000 ng/g. Sciatic nerve samples were processed using acetonitrile/water v/v 50/50 and left to shake for 1 h. Supernatant was then moved to a 96-well plate, centrifuged at 2000 rpm at 4°C for 5 min and finally injected in LC-MS/MS (Liquid chromatography-mass spectrometry/mass spectrometry).

### Niacin-SR *in vivo* treatment

Niacin-SR coated granules were resuspended prior to each administration in high viscosity methylcellulose at 0.75% (4000cP, MC, Methocel A4M, Colorcon, UK) used as vehicle and freshly prepared twice a week. Aqueous vehicle was made as following: ∼90% of the total volume of sterile deionized (DI) water was heated to ∼80°C. When temperature was reached, MC was added and then the mixture left to cool to room temperature (RT) to ensure complete MC dissolution. The final volume was ultimately reached using DI water and the solution maintained at 2–8°C protected from light.

Niacin-SR (960 mg/kg dose, once a day, gavage) was administered to mice *per os* using feeding tubes (FTPU 16-38-50, Instech Laboratories Inc., USA) from P21 until the established time-point according to the outcome. Dose of Niacin-SR coated granules was adjusted according to niacin content measured experimentally.

Animals body weight and clinical signs were closely monitored every day.

Good-practice dose-volumes guidelines (International Consortium for Innovation and Quality in Pharmaceutical Development-IQ 3Rs Leadership Group) were applied for all treatments.

### TACE activity assay

TACE activity was determined using the SensoLyte 520 TACE (α-Secretase) Activity Assay Kit *Fluorimetric* (ANASPEC, EGT Group, USA, Cat. AS-72085). After pure niacin or Niacin-SR daily treatment from P30 to P37, sciatic nerves were collected, snap-frozen in liquid nitrogen and manually pulverized with a pestle. Nerves were then homogenized using the kit assay buffer (Component c) supplemented with 0.1% Triton X-100 (SIGMA, Merck Group, USA), sonicated for 1 min, left at 4°C for 30 min on a rotating wheel and finally spin at 13.200 rpm for 15 min to collect the supernatant. Approximately 12 µg of total protein extract was loaded for each sample on a black microplate (96-well) together with TACE substrate (1:100, dilution made in assay buffer). A blank and a positive control (rh TACE, recombinant human TACE, R&D System, USA) were also included as internal controls to assess experimental reliability. The plate was then left to gently shake for 1 h at RT while preventing it from light exposure. To determine protein concentration, BCA (Bicinchoninic acid) analysis (Pierce, Thermo Fisher Scientific, USA) was performed.

Fluorescence (Excitation 490 nm, Emission 520 nm) was recorded after 1 h using a microplate reader (Victor3, PerkinElmer, UK) and then normalized for protein concentration for each sample.

### Morphological and morphometrical analysis

After collection, sciatic, quadriceps and plantar nerves were promptly fixed with 2.5% glutaraldehyde in 0.12 M phosphate buffer and left at 4°C. On the day of use, samples were first incubated with 1% osmium tetroxide (SIGMA, Merck Group, USA) for 2 h at RT to fix lipids and later embedded in epoxy resin (SIGMA, Merck Group, USA). Nerve cross sections of 1 µm were cut using Leica Ultracut UCT, stained with toluidine blue and finally displayed at a light microscope (Leica DFC300F, DE).

Images were acquired with a 100× objective and then combined to rebuild the whole section. The number of total, aberrant or degenerating fibres on total area (square millimetre) was scored using Adobe Photoshop CS4 (version 11.0) and Image J (version 1.49) software. G-ratio [axon internal diameter/fibre diameter (axon + myelin)] was instead calculated starting from five random images for each sciatic nerve at the highest magnification, 100×, using LeicaQWin software (Leica Microsystem, DE). At least 150–300 fibres per animal/nerve were counted and a total of 750–1500 fibres per group, including *n* = 5 different animals.

### Neurophysiology

2-2-2-Tribromoethanol (T48402, SIGMA, Merck Group, USA) was used as anaesthetic as previously described. Since anaesthesia induces hypothermia, mice were kept under a heating lamp during all procedures. Steel monopolar needle electrodes were placed subcutaneously near the sciatic nerve, at both proximal (at the ankle) and distal (sciatic notch) sites to obtain proximal and distal stimulation and to record motor nerve conduction velocity (NCV).

### Histology

Animals were anaesthetised with tribromoethanol (SIGMA, Merck Group, USA) as already described and then perfused with 4% paraformaldehyde. After collection, tissues were incubated O/N at RT in formalin.

The following day, samples were sectioned, placed into ethanol 70% for a minimum period of 24 h and then processed using Leica TP1020 instrument. Here, tissues undergo a scale of increasing alcoholic solutions concentration (from 70 to 100%), followed by Bioclear (BioOptica, Italy) and paraffin embedding (Bio Plast Plus, BioOptica, Italy). Samples were finally included with Leica EG1150H, cut with a rotary microtome (Leica RM2235, DE) and stained with Haematoxylin–Eosin using Leica ST5020 machine. Slides were maintained at RT until acquisition at light microscope (Leica DFC300F, DE).

### Plasma and serum collection

Approximately 500 µL of blood for each animal were collected by submandibular sampling using a hypodermic needle (Gauge G27, PIC solution) directly into tubes with Lithium Heparin (Microvette^®^, 500LH, SARSTEDT, Italy) to obtain plasma, or into 1.5 mL Eppendorf to extract serum. In both cases, samples were left at RT for 30 min and then centrifuged at 4000 rpm for 15 min. Supernatant was then collected and kept at −80°C until use.

### Bioclinical analyses

Serum analyses were performed following an International Federation of Clinical Chemistry and Laboratory Medicine optimized kinetic ultraviolet (UV) method in an ILab650 chemical analyser (Instrumentation Laboratory, USA). Urea (#0018255440), Creatinine, Crea (#0018255540), AST (#0018257540), ALT (#0018257440), Albumin (#18250040), HDL (#ch2652), LDL (#0018256040) and Cholesterol (#0018250540) were used for the quantitative determination of the serum level, and then the results expressed as milligrams per decilitre. SeraChem Control Level 1 and Level 2 (#0018162412 and #0018162512) were used as quality control.

### Plasma neurofilament light chain levels analysis

Single molecule array (SIMOA) run on an HD-X analyser (Quanterix, USA) was used to measure plasmatic levels of neurofilament light chain (NF-L) in accordance with instructions. First, samples were thawed at 21°C, vortexed, and centrifuged at 13.000 RCF (Relative centrifugal force) for 5 min at 21°C. On-board the instrument, samples were diluted 1:40 with sample diluent and bound to paramagnetic beads coated with a capture antibody specific for NF-L. NF-L bound beads were then incubated with a biotinylated NF-L detection antibody in turn conjugated to streptavidin-β-galactosidase complex that acts as a fluorescent tag. Subsequent hydrolysis reaction with a resorufin β-D-galactopyranoside substrate produces a fluorescent signal proportional to the concentration of NF-L present. Duplicate measurements were taken of each sample. Sample concentrations were extrapolated from a standard curve, fitted using a 4-parameter logistic algorithm.

### Transcriptome analysis

RNA-seq was performed for three groups of (i) control mice, (ii) *Mtmr2* KO mice and (iii) *Mtmr2* KO mice treated with Niacin-SR (*n* = 4 per genotype/condition). One sciatic nerve per mouse was collected and promptly snap-frozen with 500 μL of TRIZOL (Ambion by Life Technologies). Almost a total of 1000 ng of RNA was purified from sciatic nerve and sent to Genewiz (South Plainfield, USA) for library preparation, after PolyA selection and Illumina sequencing (Illumina HiSeq 2 × 150 bp). Illumina sequencing data were mapped to the GRCm38/mm10 genome using STAR alignment.^23^ Read counts for 10-mm transcripts were normalized and examined using DESeq2 to identify differentially regulated genes.^24,25^ The cut-off for determining significantly differentially expressed genes was an FDR (False discovery rate)-adjusted *P*-value <0.05. Gene ontology analysis was performed using Enrichr with all up-/down-regulated genes, and the Wiki Pathway Human 2023 gene sets.^26,27^ RNA-seq data and differentially expressed genes have been deposited in NCBI Gene Expression Omnibus (GSE233289). We also employed Gene Set Enrichment Analysis to analyse deregulated genes.^28,29^ Genes chosen for further analysis were cross-matched to gene expression profiling of purified Schwann cells to ensure that the reported transcripts are expressed in Schwann cells.^30^

### Immunofluorescence on frozen tissues

Tribromoethanol (SIGMA, Merck Group, USA) was used to anaesthetized animals as already described and then perfused with freshly prepared 4% paraformaldehyde (SIGMA, Merck Group, USA). Sciatic nerves were collected and immediately embedded in OCT (Compound embedding medium) (Killik, Bio-Optica, Italy) using liquid nitrogen. Both transversal and longitudinal sciatic nerve sections were cut at a cryostat with a 8 µm-thickness (Leica, DE) and left to air dry for 2 h at RT. Permeabilization was done by submerging the slides for 5 mins in ice-cold methanol or acetone (Carlo Erba, Italy). Blocking solution 1% BSA (Bovin serum albumine), 0.05% TRITON and 10% NGS (Normal goat serum) was applied for 1 h at RT before incubation with primary antibodies (2 h at RT or O/N at 4°C depending on the antigen). Sections were rinsed with PBS (Phosphate-buffered saline) 1× and then incubated for 1 h at RT with TRITC or FITC secondary antibodies (1:100). DAPI (4,6-diamidino-2-phenylindole) mounting medium (Vectashield, Vector Lab, USA) was finally used to mount slides and images acquired at Confocal Microscope SP5 (Leica, DE). Quantification of myelin basic protein (MBP) and NF fluorescence on total area (square millimetre) was performed using Image J software.

### Western blot

Sciatic nerve lysates were prepared using RIPA (Radioimmunoprecipitation assay) buffer [0.1% SDS, 0.1% Triton X-100, 100 mM NaCl, 8.4 mM Sodium phosphate dibasic (Na_2_HPO_4_), 1.6 mM Sodium phosphate monobasic (NaH_2_PO_4_) and sodium deoxycholate] to reveal LC3 and NBR1 in nerves (autophagy markers).

Standard BCA assay (Pierce, Thermo Fisher Scientific, USA) was used to determine protein concentration and 35–40 µg of proteins were loaded for each sample. Samples were run on SDS-page gels and then transferred to a pre-activated PVDF (Polyvinylidene difluoride) membrane (Millipore). Membranes were blocked overnight at 4°C with 5% Milk in PBS 0.1% Tween20 (SIGMA, Merck Group, USA) whereas both primary (anti-NBR1 and anti-LC3) and secondary antibodies [HRP (Horseradish peroxidase)-conjugated (DAKO, Agilent, USA)] were incubated in 3% milk. Immunoblots were finally developed with ECL (Enhanced luminol based detection)/ECL-prime reagents (Amersham, Merck Group, USA) using UVITEC imaging system (Cambridge, UK) and densitometry data were generated with Image J software.

### Antibodies

The following primary antibodies were used: rabbit anti-LC3 1:5000 (Sigma, #L7543); mouse anti-NBR1 1:250 (Abcam, #AB55474); mouse anti tubulin 1:2000 (Sigma, # T4026); chicken neurofilament-M 1:2000 (BioLegend, #822701); rat anti MBP 1:50 (Millipore, #MAB386); rat anti F4/80 1:200 (Biorad, #MCA497). The following secondary antibodies were used: goat anti-rabbit immunoglobulins HRP conjugated 1:10 000 (DAKO, #P0448); rabbit anti-mouse immunoglobulins HRP conjugated 1:5000 (DAKO, # P0260); donkey anti-chicken FITC conjugated 1:100 (Starfish, #703-095-155); donkey anti-rat TRITC conjugated 1:100 (Starfish, #712-025-150); donkey anti-rat FITC conjugated 1:100 (Starfish, #712-095-153).

### Statistical analysis

GPower software, v. 3.1 was used to perform power analyses as already reported.^18^

GraphPad Prism version 8 was used to perform all statistical analyses.

For each analysis, we evaluated whether the assumption required for correct application of standard parametric tests was met. In presence of two groups with *n* = 20, parametric *t*-test was used followed by Welch correction. To compare two groups with sample size smaller than *n* = 20, non-parametric Mann–Whitney *t*-test was applied.

Data from pairs of WT and mutant nerves processed in independent experiments (immunohistochemistry) were analysed using the one-sample *t*-test (non-parametric Wilcoxon test). The ratio between the mean (mean fluorescence signal of six different fields/images per nerve section) of KO values over the mean of WT (wild-type) values was calculated for each experiment and represented as a fold change in relation to WT (WT = 1). Data from pairs of WT and mutant nerves processed in independent experiments (western blot analyses) were analysed using the one-sample *t*-test (non-parametric Wilcoxon test). The ratio between the signal intensity of KO over WT was calculated for each experiment and represented as a fold change in relation to WT (WT = 1).

In presence of more than two groups, Kruskal–Wallis test, the non-parametric one-way ANOVA counterpart, was applied followed by Dunn’s *post hoc* correction.

Pearson correlation coefficient (*r*) was calculated to assess NCV and NF-L values correlation. Data are displayed as mean ± SEM and the exact *P*-value is reported in each figure legend. Results with a statistical significance have been reported in the graphs and marked as follows: *P < 0.05, **P < 0.01, ***P < 0.001 and ****P < 0.0001.

## Results

### Niacin treatment does not interfere with nerve regeneration in *Mtmr2* KO mice

**Figure 1:**
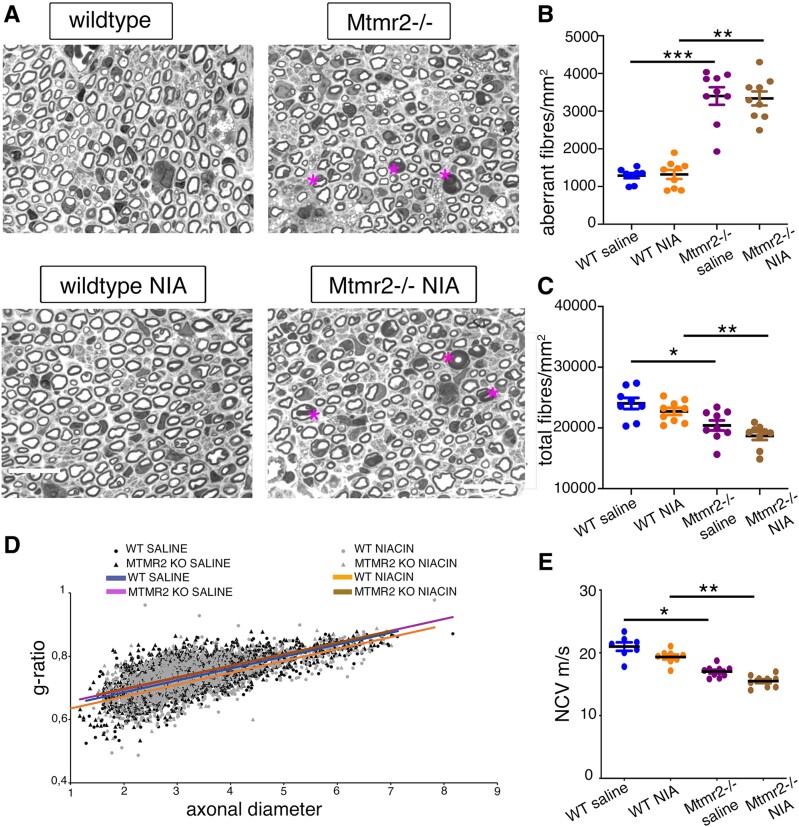
**Loss of MTMR2 impairs nerve regeneration.** (**A**) Semi-thin section analysis of the distal stump of *Mtmr2* KO sciatic nerves at 45 days post-injury (dpi) following nerve crush injury performed at P60 and daily treatment using pure niacin at 120 mg/Kg administered by intraperitoneal (i.p.) injection. Asterisks indicate aberrant fibres. (**B**) Quantification of the number of fibres per area carrying aberrant myelin in sciatic nerves at 45 dpi, non-parametric one-way ANOVA, Kruskal–Wallis test, *P* < 0.0001; Dunn’s multiple comparison test, WT saline (*n* = 8) as compared to *Mtmr2* KO saline (*n* = 9), *P* = 0.0006; WT niacin (*n* = 9) as compared to *Mtmr2* KO niacin (*n* = 9), *P* = 0.0031; WT saline (*n* = 8) as compared to WT niacin (*n* = 9), *P* > 0.9999; *Mtmr2* KO saline (*n* = 9) as compared to *Mtmr2* KO niacin (*n* = 9), *P* > 0.9999. (**C**) Quantification of the number of fibres per area in sciatic nerves at 45 dpi, non-parametric one-way ANOVA, Kruskal–Wallis test, *P* = 0.0003; Dunn’s multiple comparison test, WT saline (*n* = 8) as compared with *Mtmr2* KO saline (*n* = 9), *P* = 0.0411; WT niacin (*n* = 9) as compared with *Mtmr2* KO niacin (*n* = 9), *P* = 0.0051; WT saline (*n* = 8) as compared to WT niacin (*n* = 9), *P* > 0.9999; *Mtmr2* KO saline (*n* = 9) as compared to *Mtmr2* KO niacin (*n* = 9), *P* = 0.8233. (**D**) Morphometric analysis of sciatic nerves at 45 dpi. Distribution of *g*-ratio values, the ratio between axonal diameter (in micrometre) and fibre diameter is shown for all groups, non-parametric one-way ANOVA, Kruskal–Wallis test, *P* = 0.2219, *n* = 5 animals per group. *G*-ratio values correspond to the average of medians calculated for each group: WT saline 0.7444 ± 0.0078; *Mtmr2* KO saline 0.7401 ± 0.0066; WT niacin 0.7310 ± 0.0038; *Mtmr2* KO niacin 0.739 ± 0.0027. WT saline (*n* = 5) as compared with *Mtmr2* KO saline (*n* = 5), *P* > 0.9999; WT niacin (*n* = 5) as compared with *Mtmr2* KO niacin (*n* = 5), *P* > 0.9999; WT saline (*n* = 5) as compared to WT niacin (*n* = 5), *P* = 0.1918; *Mtmr2* KO saline (*n* = 5) as compared to *Mtmr2* KO niacin (*n* = 5), *P* > 0.9999. (**E**) NCV analysis at 45 dpi, non-parametric one-way ANOVA, Kruskal–Wallis test, *P* < 0.0001; Dunn’s multiple comparison test, WT saline (*n* = 7) as compared with *Mtmr2* KO saline (*n* = 9), *P* = 0.0103; WT niacin (*n* = 8) as compared with *Mtmr2* KO niacin (*n* = 9), *P* = 0.0012; WT saline (*n* = 7) as compared to WT niacin (*n* = 8), *P* > 0.9999; *Mtmr2* KO saline (*n* = 9) as compared to *Mtmr2* KO niacin (*n* = 9), *P* = 0.3164. Data are mean ± SEM. WT NIA and Mtmr2^−/−^ NIA refer to animals treated with pure niacin. Bar in **A** is 17.55 μm.

NRG1 type III is required for remyelination after injury, similarly to NRG1 type I, another NRG1 isoform released by Schwann cells and necessary for nerve regeneration.^20-22^ Thus, we explored whether downregulation of NRG1 signalling via niacin-mediated TACE activation might be detrimental to nerve regeneration. To this aim, we performed nerve crush injury experiments in *Mtmr2* KO mice at P60, which are characterized by aberrant myelin in the nerve and reduced NCV, thus reproducing features of CMT4B1.^8,9^ Starting from the day after nerve crush, we treated both WT and mutants with niacin administered daily by i.p. injection at 120 mg/Kg as already reported.^18^ After 45 dpi (days post-injury) of consecutive treatment, we performed morphological and morphometric analyses of distal stumps of sciatic nerves (Fig. 1A–D). We first observed that the number of regenerating fibres (total number of fibres) in *Mtmr2* KO nerves was significantly decreased as compared with WT (Fig. 1A and C) and that the number of fibres carrying aberrant myelin was increased in mutant nerves as compared with WT after crush injury (Fig. 1A and B). The *g*-ratio, the ratio between axon diameter and fibre diameter (myelin plus axon) was similar between WT and *Mtmr2* KO injured nerves, as already reported in intact nerves (Fig. 1D). Neurophysiological analysis showed reduction of NCV values in injured *Mtmr2* KO nerves as compared with controls, thus further suggesting a regeneration defect in this model (Fig. 1E). Importantly, niacin treatment had no effect in either WT or *Mtmr2* KO nerves, suggesting that, despite reducing indispensable signals mediated by NRG1 type III in the axon and/or NRG1 type I in Schwann cells, niacin is not detrimental to nerve regeneration (Fig. 1A–E). To confirm niacin-mediated TACE activation, we measured TACE activity from both intact and crushed nerves of control mice treated for seven days with niacin. As expected we found that TACE activity was increased in intact nerves but not in crushed nerves at 7 dpi, when axons which express NRG1 type III rapidly degenerate (Supplementary Fig. 1A and B in Supplementary_material_1). Altogether, these results indicate that the loss of MTMR2 alters nerve regeneration and that niacin treatment is not detrimental to nerve regeneration.

**Figure 2:**
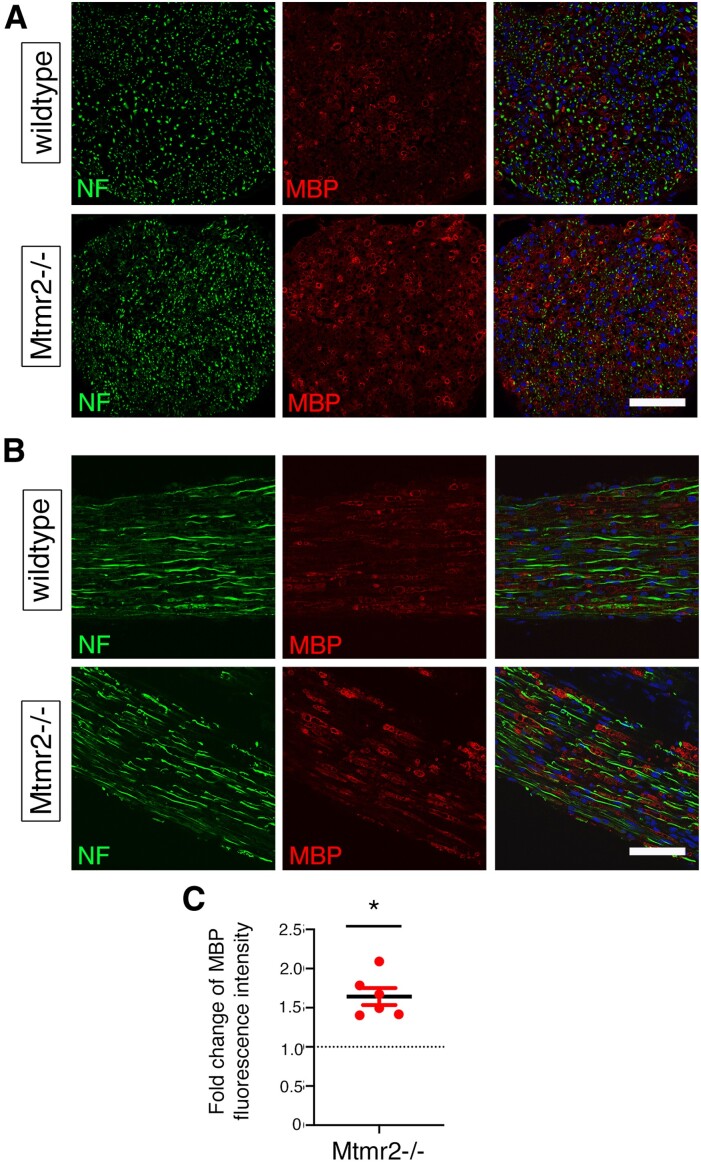
**Loss of MTMR2 delays myelin clearance.** (**A**) Immunohistochemistry at 7 days post-injury (dpi) of distal stump of sciatic nerves following nerve crush injury performed at P60 in transverse (**A**) and longitudinal sections (**B**). (**C**) Quantification of the MBP signal intensity per area in transverse and in longitudinal nerve sections expressed as fold change to WT set to 1, two-tailed, Wilcoxon one-sample *t*-test, *n* = 6 animals analysed per genotype in six independent experiments (*n* = 3 transverse and *n* = 3 longitudinal sections analysed per genotype, as described in Statistical methods), *P* = 0.0312. Data are mean ± SEM. Bar in **A** is 114 μm and in **B** is 110 μm. MBP, myelin basic protein; NF, neurofilament.

**Figure 3:**
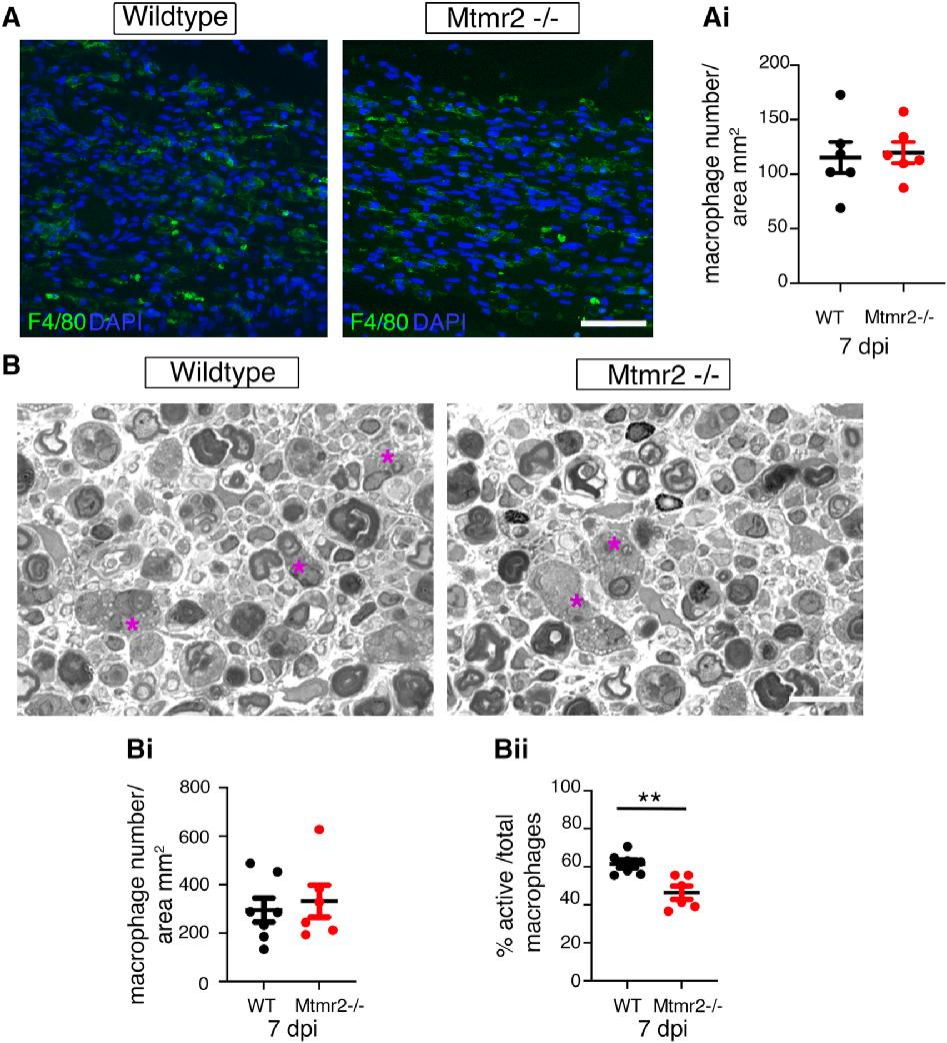
**Macrophage analysis in *Mtmr2* KO crushed nerves.** (**A**) Immunohistochemistry using the F4/80 marker to detect macrophages in *Mtmr2* KO crushed nerves and controls at 7 days post-injury (dpi). (**Ai**) *Mtmr2* KO and controls nerves show a similar number of macrophages, two-tailed, Mann–Whitney *t*-test, *n* = 6 animals analysed per genotype in six different experiments, *P* = 0.4848. Each value, which is the number of F4/80 positive cells per area, represents the mean of values obtained by counting 10–20 images per nerve section/animal. (**B**) Semi-thin section analysis of distal stumps of sciatic nerves at 7 dpi, with asterisks marking ‘active’ foamy macrophages. Mutant and control nerves show a similar number of macrophages quantified in **Bi**, whereas active macrophages were significantly decreased in *Mtmr2* KO nerves. (**Bi**) *n* = 7 WT and *n* = 6 *Mtmr2* KO animals, two-tailed Mann–Whitney *t*-test, *P* = 0.731. (**Bii**) *n* = 7 WT and *n* = 6 *Mtmr2* KO animals, two-tailed Mann–Whitney *t*-test, *P* = 0.0023. Data are mean ± SEM. Bar in **A** is 46 μm, in **B** is 13.28 μm.

To better characterize the regeneration defect observed in *Mtmr2* KO nerves, we performed nerve crush injury experiments in WT and *Mtmr2* KO mice at P60 and analysed the distal stump at 7 dpi. By performing immunohistochemistry on both transverse (Fig. 2A) and longitudinal (Fig. 2B) sections of distal sciatic nerve stumps, we found that MBP expression per area was increased in *Mtmr2* KO nerves as compared with WT (Fig. 2C). This finding suggests a delay in myelin clearance which, in the first 2–5 days post-injury, is dependent on both Schwann cell-mediated autophagy and macrophage-mediated phagocytosis.^31^ Of note, MTMR2 preferentially dephosphorylates the PtdIns(3,5)*P*_2_ phosphoinositide, which regulates late endosomal–lysosomal associated functions, and particularly autophagic progression.^32,33^ We thus investigated autophagy in *Mtmr2* KO sciatic nerves at both 3 and 7 dpi post-crush injury. Expression of LC3II/I, which is a maker of autophagy induction, and of NBR1, a marker of autophagy progression, were similar in WT and *Mtmr2* KO crushed nerves at both time-points analysed (Supplementary Figs. 2A, B′, 7 and 8 in Supplementary_material_1). We then explored macrophage number in *Mtmr2* KO crushed sciatic nerves. To this aim, we performed immunohistochemistry on distal stumps of WT and *Mtmr2* KO nerves at 7 dpi using F4/80, a macrophage marker. The number of macrophages per area was similar in the two genotypes (Fig. 3A and Ai). This finding was further confirmed by semi-thin section analyses of sciatic nerves at 7 dpi (Fig. 3B and Bi). On the contrary, the number of ‘active macrophages’ defined as cells carrying extensive cytoplasm filled by vacuoles and myelin debris were significantly decreased in *Mtmr2* KO crushed nerves, suggesting altered macrophage-mediated clearance (Fig. 3B and Bii).

### Extended-release formulation of niacin

We already provided evidence that niacin-mediated modulation of TACE activity can be effective to ameliorate CMT neuropathies associated with excessive aberrant myelin.^18^ We obtained proof-of-principle of this strategy in different models of hypermyelination and aberrant myelin including *Mtmr2* KO mice. We previously treated these mouse models using 120 mg/Kg of niacin by i.p. injection daily for 45 days starting at P15 and morphological analysis of sciatic nerves was performed at the end-point. In *Mtmr2* KO mice, decreased NCV can be measured starting at 5–6 months of age. Thus, to assess whether niacin can ameliorate nerve physiology, long-term preclinical trials must be performed using this model. To this aim, we treated *Mtmr2* KO mice for five consecutive months using an extended-release formulation of niacin administered by oral gavage, thus mimicking Niaspan^®^ in human. A prolonged release of niacin was preferred compared to an immediate delivery of the drug to avoid peaks of concentration, which might exert toxicological effects, and to induce a prolonged duration of the effective concentration. Niaspan^®^, the FDA-approved medicinal product, is based on tablet compositions containing 500, 750 or 1000 mg of niacin along with excipients able to control the release of the drug for ∼24 h. Since oral administration of extended-release tablets to mice was not feasible, reservoir systems for sustained release of niacin were prepared by coating granules with a controlling membrane based on ethylcellulose as the main insoluble polymer and hypromellose as the pore former. Size of the coated beads were limited to 500 µm for avoiding clogging of oral gavage tubes selected for the oral administration to mice having internal diameter 0.8 mm. *In vitro* drug release from coated granules was customized by controlling the coating formulation and amount to match that of Niaspan^®^. For a more accurate dosing, coated granules were suspended in a relatively viscous aqueous solution (0.75% methylcellulose), which prevented sedimentation and allowed for complete transfer of granules from the dosing syringe. Dose of Niacin-SR beads was adjusted according to niacin content.

**Figure 4:**
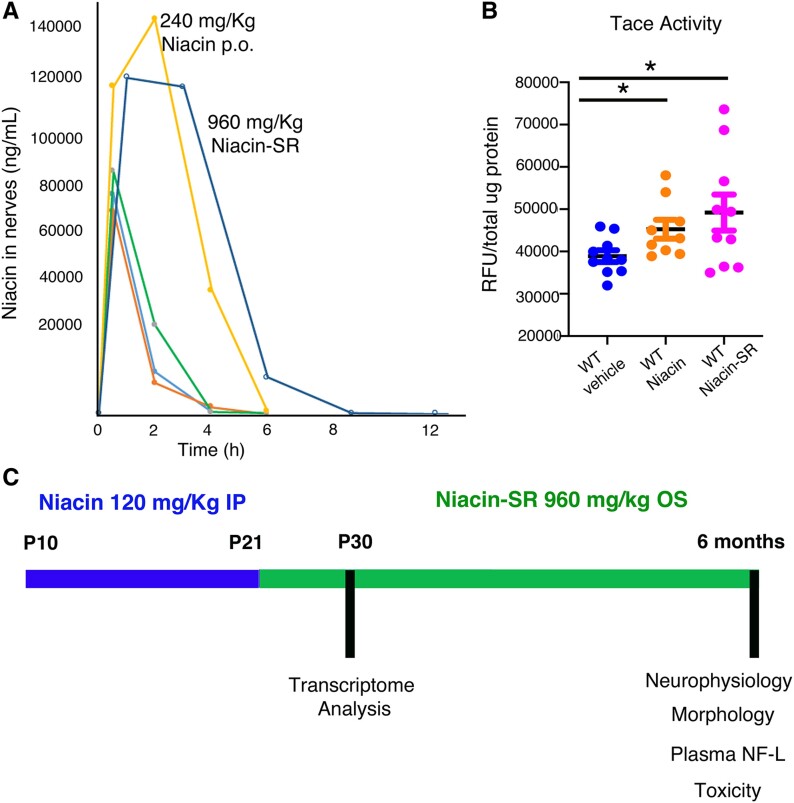
**Preclinical trial in *Mtmr2* KO mice using Niacin-SR.** (**A**) Pharmacokinetic profile of niacin concentration in nerves from control mice treated daily for 1 week using Niacin-SR 960 mg/Kg *per os* (blue line), pure niacin *per os* at 240 mg/Kg (yellow line), Niacin-SR 720 mg/Kg *per os* (green line), pure niacin administered by i.p. injection and *per os* at 120 mg/Kg (orange and light blue lines). (**B**) TACE activity measured from sciatic nerve lysates of control mice treated for 1 week daily with vehicle, pure niacin administered by i.p. injection at 120 mg/Kg and Niacin-SR administered by gavage *per os* at 960 mg/Kg, non-parametric one-way ANOVA, Kruskal–Wallis test, *P* = 0.065; Dunn’s multiple comparison test WT vehicle (*n* = 10) as compared with either WT niacin (*n* = 9) *P* = 0.0471 or WT Niacin-SR (*n* = 10), *P* = 0.0405, whereas WT niacin (*n* = 9) as compared with WT Niacin-SR (*n* = 10) did not show statistical differences, *P* = 0.9932. Data are mean ± SEM. (**C**) Schematic view of the preclinical trial performed in *Mtmr2* KO mutants, IP, intraperitoneal injection; OS, oral gavage administration; Niacin-SR, slow release; NF-L, neurofilament light chain; TACE, TFN-α convertase enzyme; RFU, relative fluorescence unit.

To evaluate the dosage of Niacin-SR to be administered daily *per os*, we initially treated WT mice starting at P30 for seven consecutive days by daily i.p or oral gavage in parallel using both 120 and 240 mg/Kg of pure niacin. Then, we measured niacin concentration from plasma and nerves at different time-points such as 0, 4, 6, 8 and 12 h after the last administration. As shown in Fig. 4A, the concentration of niacin in the nerve was similar between 120 mg/Kg i.p. and 120 mg/Kg *per os*, whereas it was significantly increased using 240 mg/Kg of niacin *per os*, which, however, was rapidly metabolized after 4 h. On the contrary, we found that 960 mg/Kg of Niacin-SR administered daily by oral gavage for seven consecutive days recapitulated the kinetic profile of 240 mg/Kg pure niacin but lasting for 6–8 h in the tissue.

To confirm that this formulation and dosage were effective in increasing TACE activity, we measured TACE activity from sciatic nerves of WT animals treated from P30 for seven consecutive days using either 960 mg/Kg of Niacin-SR administered daily by oral gavage or with 120 mg/Kg pure niacin administered by i.p., as a positive control. We observed that Niacin-SR significantly increased TACE activity in the nerve even more efficiently than pure niacin solution (Fig. 4B).

### Niacin-SR ameliorates CMT4B1 neuropathy in *Mtmr2* KO mice

We thus treated *Mtmr2* KO mice and controls using 960 mg/Kg of Niacin-SR daily by oral gavage starting at P21 until P180 (*n* = 14 WT vehicle; *n* = 10 WT Niacin-SR; *n* = 24 *Mtmr2* KO vehicle, and *n* = 24 *Mtmr2* KO Niacin-SR treated enrolled). Given the size of the cellulose beads and the diameter and size of the tip used, oral gavage was performed starting from P21 (Fig. 4C). However, since myelin outfoldings and aberrant myelin are already present in mutant nerves from the first week of postnatal development, we also treated *Mtmr2* KO mice by performing daily i.p. injection from P10 to P21 using pure niacin at 120 mg/Kg (Fig. 4C).

**Figure 5:**
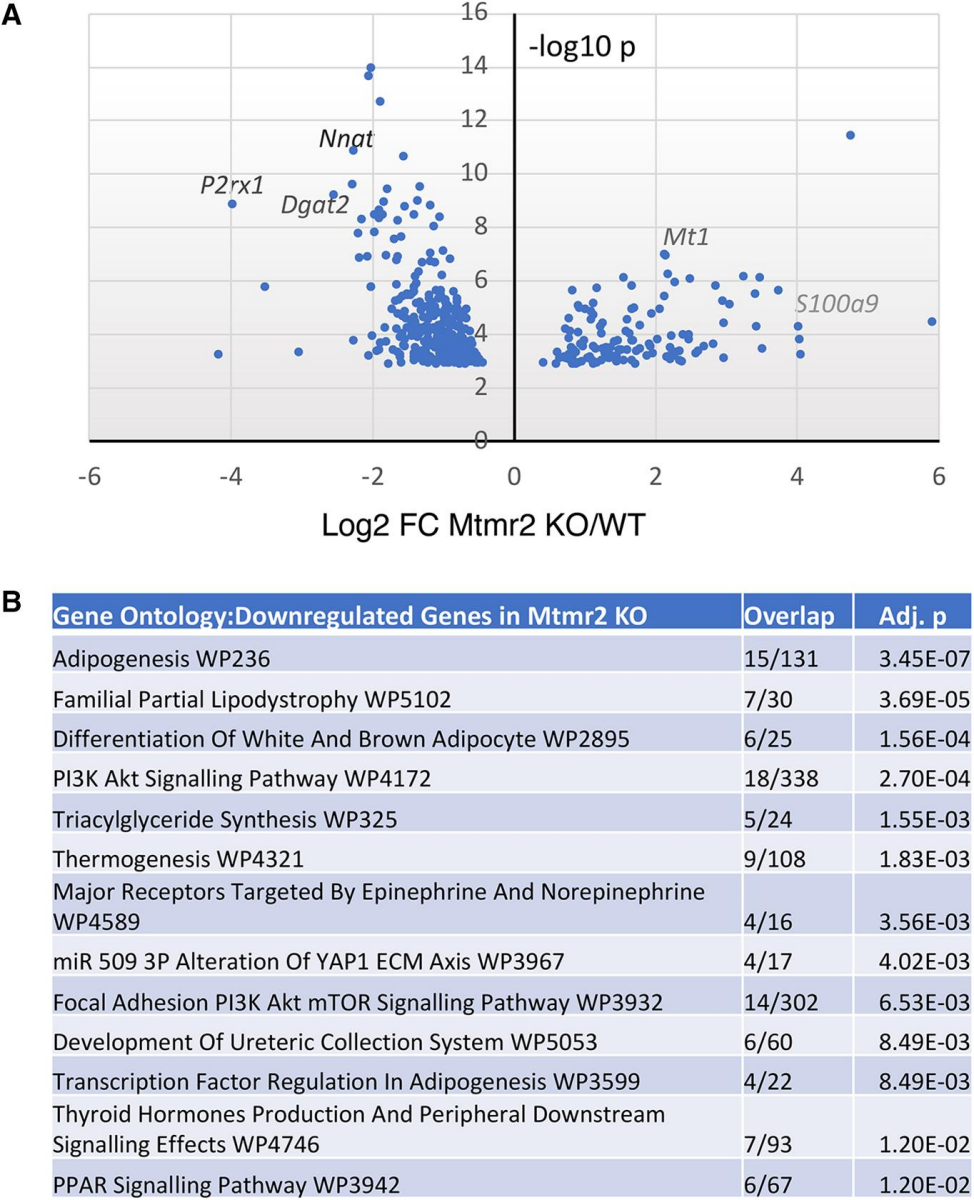
**RNA-Seq analysis of *Mtmr2* KO mouse sciatic nerves.** (**A**) Gene expression profiles are shown as a volcano plot of log2-fold change versus −log10 *P*-value. Significantly deregulated genes include elevated genes such as *S100a9* and *Mt1*, and downregulated genes (*Nnat*, *Dgat2* and *P2rx1*). (**B**) Gene ontology analysis of downregulated genes indicate enrichment of several categories, including some related to PI3K–AKT and PPAR signalling. The top enriched pathways (Wiki Pathways Human 2023) are shown. AKT, V-Akt murine thymoma viral oncogene-like protein; PI3K, phosphatidyl inositol 3 kinase; mTOR, mechanistic target of rapamycin kinase, PPAR, peroxisome proliferator activated.

To assess whether the treatment was effectively reaching the nerve, we determined the gene expression changes in the *Mtmr2* KO mice as compared to control and *Mtmr2* KO Niacin-SR treated by performing RNA-seq analysis from sciatic nerves at P30 (Fig. 4C). First, comparison of the *Mtmr2* KO mice to WT controls (*n* = 4) revealed a significant number of changes as shown in the volcano plot (Fig. 5A). One hundred and thirty-five genes were increased in *Mtmr2* KO and 297 genes were decreased compared to control (adj *P* < 0.05). Gene ontology analysis was employed to analyse up- and down-regulated genes that reached statistical significance (*P* < 0.05). One of the notable aspects of the analysis was several enriched gene categories among downregulated genes (Fig. 5B and Supplementary_material_3). These included categories related to lipid synthesis, AKT, PPAR and mTOR pathways, which have been implicated in NRG1 signalling.^14,34-40^ Gene set enrichment analysis also identified lipid biosynthetic gene categories among downregulated genes in the *Mtmr2* KO (Supplementary_material_2).^28,29^

**Figure 6:**
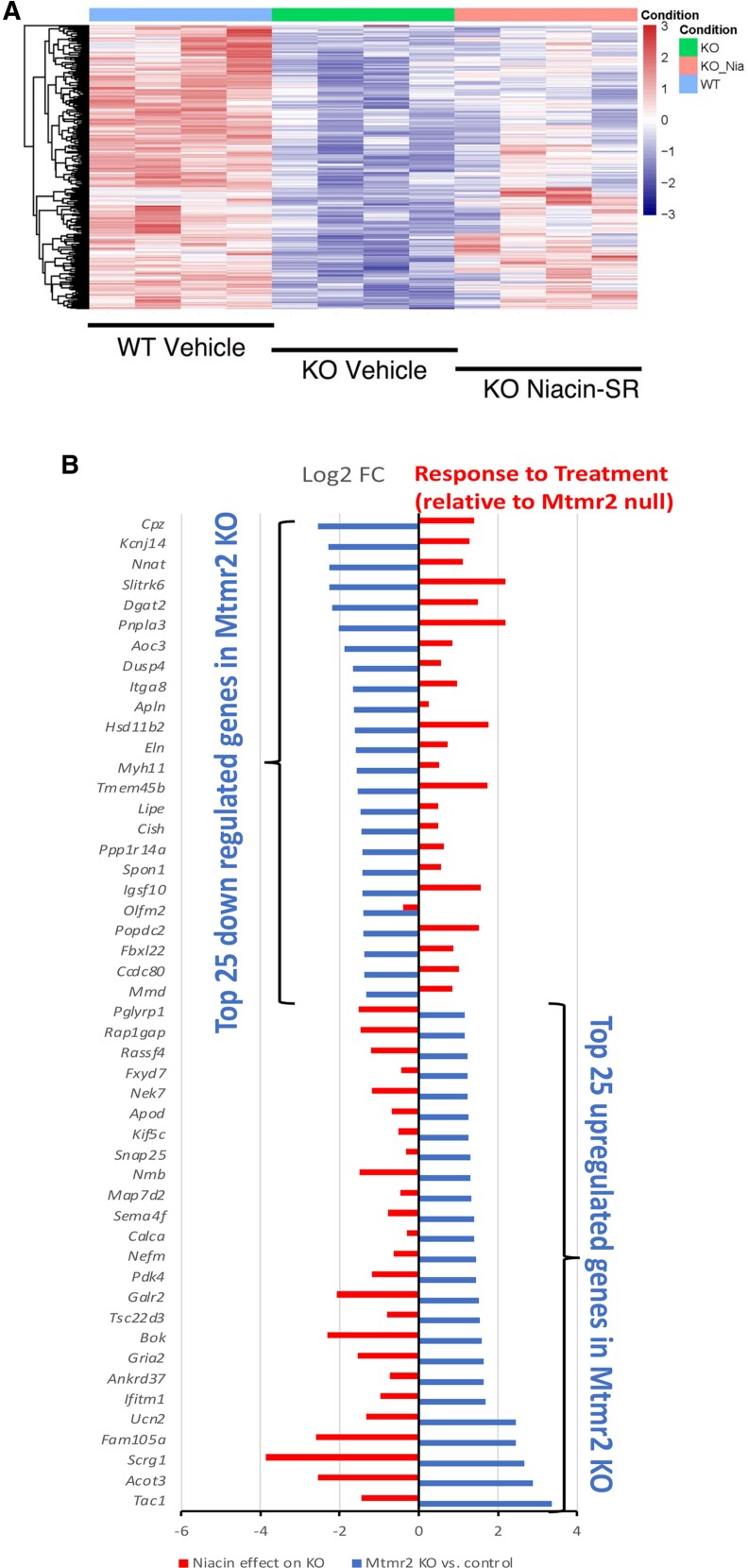
**Treatment effect of niacin in the *Mtmr2* KO.** (**A**) The heat map indicates the relative expression for each nerve sample (*n* = 4) within the three groups: control, *Mtmr2* KO, and niacin-treated *Mtmr2* KO sciatic nerves. The heat map shows genes downregulated in the *Mtmr2* KO samples. (**B**) The figure shows the top 25 significantly regulated genes (*P* < 0.05), which are either down- or up-regulated in the *Mtmr2* KO (blue bars, log2-fold change). For each of these genes, the change in the niacin-treated *Mtmr2* KO group relative to the *Mtmr2* KO is shown by the red bars, indicating that most gene level changes in the *Mtmr2* KO are reversed by niacin treatment.

We also assessed if the niacin treatment group reversed any of the changes in the *Mtmr2* KO, which yielded 245 genes increased by niacin treatment of the *Mtmr2* KO mice and 198 genes decreased by niacin treatment (adj *P* < 0.05, Supplementary Fig. 3 in Supplementary_material_1). We first filtered the gene expression changes using a recently published analysis of sorted Schwann cells.^30^ Fig. 6A shows a heat map of the genes downregulated in the *Mtmr2* KO, and the response of these genes to treatment. In Fig. 6B, virtually all of the top 25 upregulated/downregulated genes (adj *P* < 0.05) in the *Mtmr2* KO mice compared to control were reversed in the niacin treatment group (niacin treatment *Mtmr2* KO compared with *Mtmr2* KO vehicle treated), including several genes involved in lipid synthesis, such as *Srebf1*, *Scd1*, *Lpl* and *Pparg*. These results are strongly suggestive of a treatment response in *Mtmr2* KO sciatic nerves.

Niacin-SR treatment was well tolerated as shown by biochemical and histological analyses performed at the end-point, at P180. Several blood markers monitoring liver and kidney function, the two main organs where niacin is catabolized showed similar levels between WT and *Mtmr2* KO mice treated with vehicle or Niacin-SR (Supplementary Fig. 4 in Supplementary_material_1). Moreover, gross histological analysis of gut, liver and kidney did not reveal signs of inflammation or tissue damage in both WT and *Mtmr2* KO treated with Niacin-SR at 6 months of age (Supplementary Fig. 5 in Supplementary_material_1).

**Figure 7:**
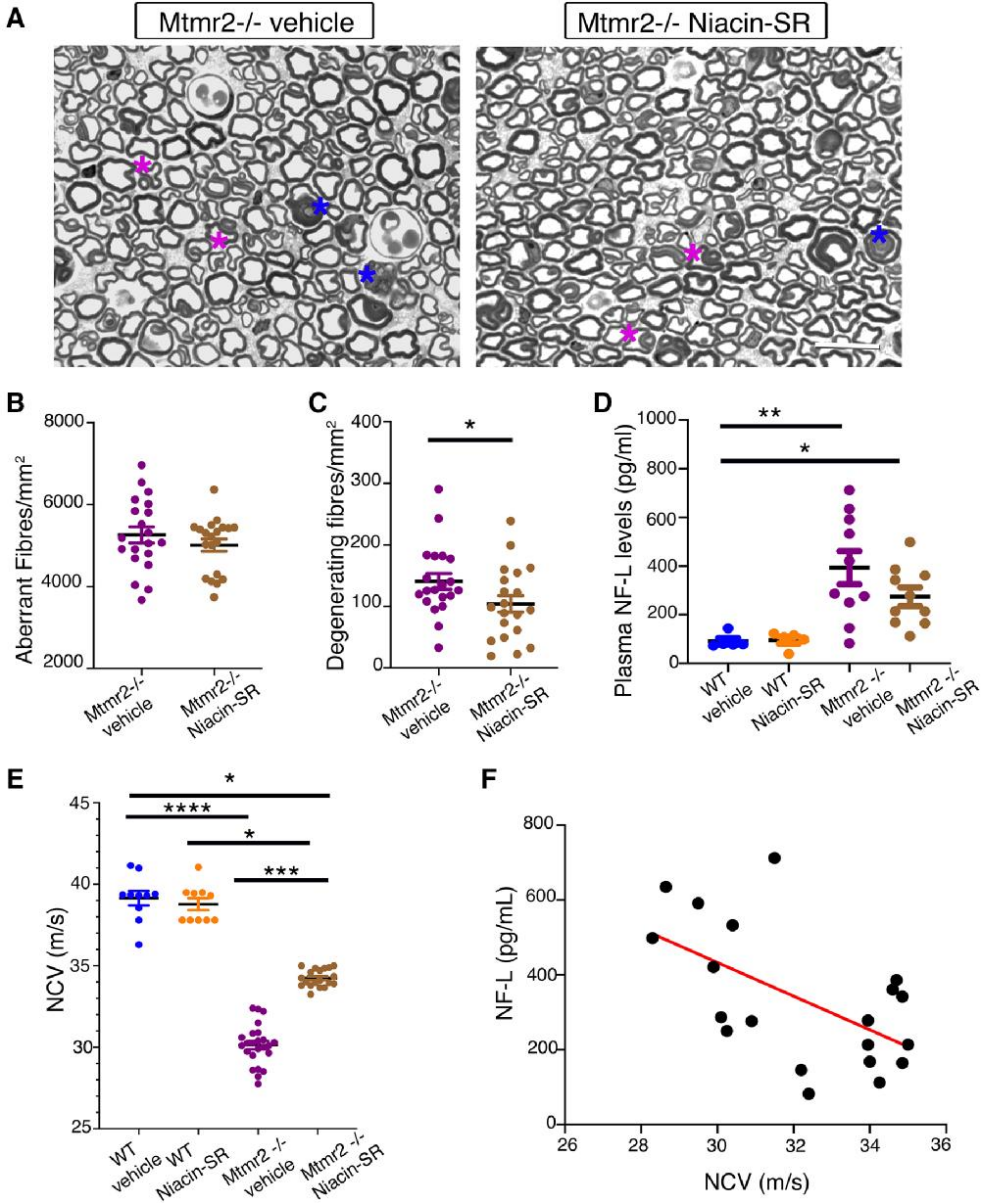
**Outcome measures analyses in *Mtmr2* KO mice treated using Niacin-SR until 6 months of age.** (**A**) Semi-thin section analysis of sciatic nerves from *Mtmr2* KO mice treated with vehicle or Niacin-SR as shown in Fig. 4. Blue asterisks indicate degenerating fibres whereas pink myelin outfoldings. (**B**) Quantification of the total number of aberrantly myelinated fibres (outfoldings, tomacula, etc.) per nerve section/area in *Mtmr2* KO vehicle-treated (*n* = 20 mice) and *Mtmr2* KO Niacin-SR treated (*n* = 20 mice), one-tailed Welch’s *t*-test, *P* = 0.1605, *t* = 1.007, df = 35.61. (**C**) Quantification of the total number of degenerating fibres per nerve section/area in *Mtmr2* KO vehicle-treated (*n* = 20 mice) and *Mtmr2* KO Niacin-SR treated (*n* = 20 mice), one-tailed Welch’s *t*-test, *P* = 0.0278, *t* = 1.975, df = 37.96. Welsch’s test was applied given the sample size and assuming normal distribution, whereas one-tailed was used assuming that niacin can either improve or have no effect on the phenotype.^18^ (**D**) Quantification of the plasma levels of NF-L from WT mice treated with vehicle or Niacin-SR (*n* = 5 mice) and from *Mtmr2* KO mice treated with vehicle (*n* = 10) or Niacin-SR (*n* = 10), non-parametric one-way ANOVA, Kruskal–Wallis test, *P* = 0.0007; Dunn’s multiple comparison test, WT vehicle as compared with *Mtmr2* KO vehicle, *P* = 0.0034; WT vehicle as compared with *Mtmr2* KO Niacin-SR *P* = 0.0273; WT vehicle as compared with WT Niacin-SR, *P* > 0.9999, *Mtmr2* KO vehicle as compared with *Mtmr2* KO Niacin-SR, *P* > 0.9999; WT Niacin-SR as compared with *Mtmr2* KO Niacin-SR *P* = 0.1010. (**E**) NCV values in the four groups WT vehicle, 39.15 m/s (*n* = 10 mice); WT Niacin-SR, 38.78 m/s (*n* = 10 mice); *Mtmr2* KO vehicle, 30.13 m/s (*n* = 24) and *Mtmr2* KO Niacin-SR, 34.24 m/s (*n* = 19), non-parametric one-way ANOVA, Kruskal–Wallis test, *P* < 0.0001; Dunn’s multiple comparison test WT vehicle as compared with *Mtmr2* KO vehicle, *P* < 0.0001; WT Niacin-SR as compared with *Mtmr2* KO Niacin-SR *P* = 0.0405; *Mtmr2* KO vehicle as compared with *Mtmr2* KO Niacin-SR, *P* = 0.0007; WT vehicle as compared with WT Niacin-SR *P* > 0.9999; WT vehicle as compared with *Mtmr2* KO Niacin-SR, *P* = 0.0254. (**F**) Correlation analysis between NCV and plasma levels of NF-L, *P* = 0.0083, *r* = −0.573, *n* = 20, including both *Mtmr2* KO vehicle and *Mtmr2* KO Niacin-SR treated. NF-L is the neurofilament light chain. Data are mean ± SEM. Bar in **A** is 13 μm.

**Figure 8:**
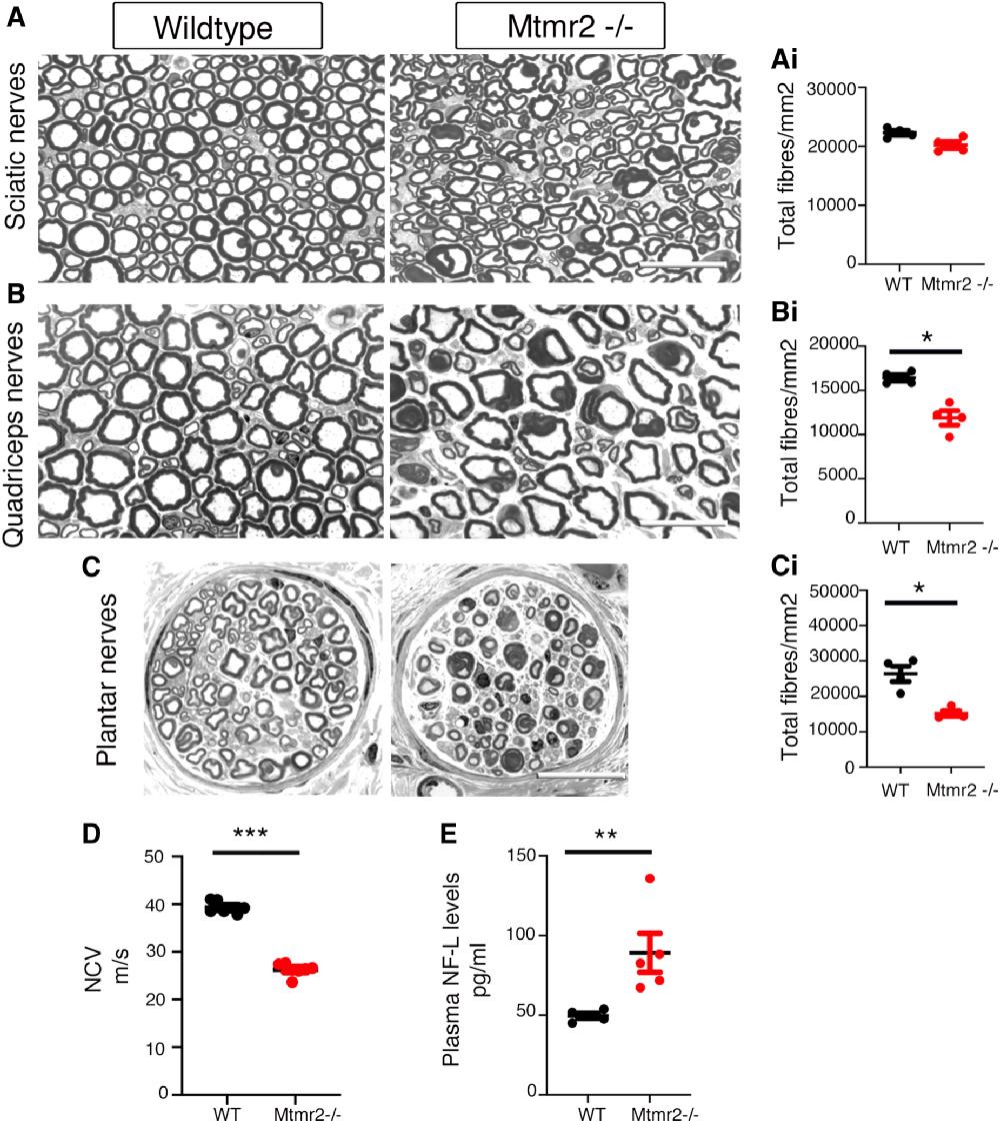
**Fibre loss in nerves from *Mtmr2* KO mice at 12 months.** (**A**) Semi-thin section analysis of WT and mutant sciatic nerves with quantification of the total number of fibres per nerve section (**Ai**), two-tailed Mann–Whitney *t*-test, *P* = 0.0571, *n* = 4 animals per genotype. (**B**) Semi-thin section analysis of WT and mutant quadriceps nerves with quantification of the total number of fibres per nerve section (**Bi**), two-tailed Mann–Whitney *t*-test, *P* = 0.029, *n* = 4 animals per genotype. (**C**) Semi-thin section analysis of WT and mutant plantar nerves with quantification of the total number of fibres per nerve section (**Ci**), two-tailed Mann–Whitney *t*-test, *P* = 0.029, *n* = 4 animals per genotype. (**D**) NCV measured in WT and mutant mice at 12 months of age, WT, 39.3 m/s and Mtmr2 KO, 26.27 m/s, two-tailed Mann–Whitney *t*-test, *P* = 0.0006, *n* = 7 animals per genotype. (**E**) Plasma NF-L levels in WT and mutant mice at 12 months, two-tailed Mann–Whitney *t*-test, *P* = 0.0079, *n* = 5 animals per genotype. Data are mean ± SEM. Bar in **A** is 19.8 μm; in **B** 22.03 μm and in **C** 29.75 μm.

To further assess efficacy of this strategy, neurophysiological analyses was performed at the end-point, at 6 months of age. We found that NCV, which is decreased in *Mtmr2* KO mice, was significantly increased in niacin/Niacin-SR treated mutants (Fig. 7E). Semi-thin section analyses of sciatic nerves showed that aberrant fibres were not ameliorated by the treatment but, interestingly, the number of degenerating fibres was slightly reduced in *Mtmr2* KO mice treated with Niacin-SR as compared with *Mtmr2* KO (Fig. 7A–C and Supplementary Fig. 6 in Supplementary_material_1). Of note, the total number of fibres is similar between mutant and controls sciatic nerves at 6 months of age (*n* = 9 WT vehicle, 2259 ± 41.03; *n* = 9 WT Niacin-SR, 2292 ± 40.25; *n* = 20 *Mtmr2* KO vehicle, 2307 ± 14.66; *n* = 20 *Mtmr2* KO Niacin-SR, 2315 ± 24.59). We also measured plasma levels of NF-L, neurofilament light chain, a marker of axonal degeneration in CMT neuropathies in both patients and mice.^41^ NF-L was slightly increased in *Mtmr2* KO plasma as compared with WT mice whereas Niacin-SR treatment reduced its level in the mutant although not significantly (Fig. 7D). Finally, we found that the NF-L levels inversely correlated with NCV values, as higher NF-L suggestive of fibre degeneration correlated with lower NCV values and vice versa (Fig. 7F). To explore whether fibre degeneration and increased NF-L levels are indicative of fibre loss at later time-points, we analysed *Mtmr2* KO nerves at 12 months. Indeed, we found that the total number of fibres in sciatic, quadriceps and plantar nerves was significantly reduced in *Mtmr2* KO as compared with controls. Importantly, fibre loss observed at 12 months correlated with a further increase in NF-L plasma levels in *Mtmr2* KO at 12 months (Fig. 8). In conclusion, these findings further suggest that Niacin-SR treatment ameliorates nerve physiology and may preserve fibres from degeneration.

## Discussion

NRG1 type III is one of the main factors regulating PNS development.^14-16^ NRG1 type III on the axonal surface dictates if an axon will be myelinated and the amount of myelin produced by Schwann cells, which is a function of the axonal diameter. BACE and TACE secretases positively and negatively regulate NRG1 type III function, respectively.^17^ Of note, niacin, nicotinic acid is a TACE activator and Niaspan^®^, an extended-release formulation of niacin, is a FDA-approved drug used to treat dyslipidaemia in human.^42-44^ NRG1 type III is dispensable for myelin maintenance, whereas it is required for nerve regeneration following injury. NRG1 type I is another NRG1 isoform that is mainly expressed by Schwann cells. Whilst it is dispensable for myelination, NRG1 type I is instead crucial to guide Schwann cells during early stages of nerve regeneration.^20-22^

CMT4B1 is a severe autosomal recessive neuropathy characterized by excessive aberrant myelin in the nerve.^6^ This morphology is similar to that observed in nerves of other CMT forms such as CMT4B2, B3, CMT4H and HNPP, the hereditary liability to pressure palsies.^5^ We recently explored efficacy of the niacin-mediated TACE activation as a strategy to decrease NRG1 type III signalling and ameliorate CMT neuropathies associated with excessive redundant myelin in the nerve. We provided proof-of-concept of this strategy using different models of hypermyelination, including the *Mtmr2* KO mouse, which recapitulates CMT4B1.^18^ In the perspective of a clinical translation of this strategy, here we aimed at assessing whether niacin-mediated TACE activation and reduction of NRG1 type III ameliorates the phenotype of neuropathic models without interfering with nerve regeneration, which is dependent on this signalling. We thus performed nerve crush injury experiments using the *Mtmr2* KO model and we found that niacin administration was not detrimental for nerve regeneration. This is extremely important as CMT4B neuropathy is also characterized by axonal damage, and any potential treatment should not interfere with possible axonal regeneration. Surprisingly, we observed that *Mtmr2* KO have a defect in nerve regeneration, which was not previously reported. *Mtmr2* KO crushed nerves displayed fewer regenerating fibres and more fibres carrying aberrant myelin at 45 dpi as compared to controls. Importantly, NCV values were reduced at 45 dpi in *Mtmr2* KO crushed nerves. We also showed that this defect may be the consequence of a delay of myelin clearance, which is a prerequisite for regeneration. As myelin removal in the PNS depends on both macrophage-mediated phagocytosis and Schwann cell-mediated autophagy at 3–5 dpi,^31^ we investigated these processes in *Mtmr2* KO nerves at 3 and 7 dpi. Autophagic flux was similar between mutant and control nerves, whereas the number of active foamy macrophages was reduced in *Mtmr2* KO nerves, which may result in a delay of clearance. However, further experiments are necessary to corroborate this conclusion.

Fibre degeneration, axon loss and reduced conduction are at the basis of clinical disability in CMT4B1 patients. Neurophysiology is informative in the *Mtmr2* KO model starting from 6 months of age and thus long-term preclinical trials are necessary to assess efficacy of niacin-mediated therapy at the functional level. To this aim, we treated *Mtmr2* KO mice by oral gavage for five consecutive months using niacin. Niaspan^®^ is the FDA-approved medicinal product with a long-lasting effect at 24 h in humans. A prolonged release of niacin is preferred as compared to an immediate delivery of the drug to avoid peaks of concentration, which might exert toxicological effects, and to induce a prolonged duration of the effective concentration. To mimic the long-lasting effect of human Niaspan^®^, we prepared reservoir systems for sustained release of niacin to be administered to mice by oral gavage and we coated granules with a controlling membrane based on ethylcellulose as the main insoluble polymer and hypromellose as the pore former. This formulation, Niacin-SR, was well tolerated by mice when administered *per os* daily at 960 mg/Kg and it was effective in enhancing TACE activity in the nerve. More important, our data showed that this treatment was able to revert the abnormal transcriptomic profile of *Mtmr2* KO nerves at P30. When outcome measures were scored at 6 months, the end-point of this trial, we observed that treated mutant mice had a significant increase in NCV as compared with vehicle-treated *Mtmr2* KO and a decrease in the number of degenerating fibres. We also noted that plasma NF-L levels, a marker of neurodegeneration, slightly decreased following Niacin-SR treatment. Importantly, there was a significant inverse correlation between levels of NF-L and NCV values, with mice showing higher NCV that had reduced NF-L levels. We further confirmed that increased NF-L levels and fibre degeneration are preceding fibre loss in this model, which can be observed at 12 months in different nerves.

However, differently to our previous report,^18^ the number of fibres carrying aberrant myelin, myelin outfoldings, was similar between *Mtmr2* KO mice treated with Niacin-SR as compared with vehicle treated mutants. Whether the drug partially ameliorated the dys-myelinating phenotype earlier at 2 months following treatment thus reproducing previous findings is not known. Of note, pure niacin administration via i.p. injection in the *Fdg4* KO (FRABIN) mouse at P30 for 8 weeks, a model of CMT4H also characterized by myelin outfoldings, was able to reduce the number of myelin alterations.^45^ Alternatively, we can speculate that the formulation used and the different delivery (*per os* in this study as compared to i.p. injection in previous study) may result in different effects due to the different pharmacokinetic and biodistribution.^46^ This is not novel for niacin, that is known to have different effects depending on dose and formulation.^19^ Niacin and Niaspan^®^ have been used for many years as anti-dyslipidaemic drugs. In addition, niacin through its receptor GPR109A (HCAR2) highly expressed in monocytes/macrophages, has been found to increase microglia-mediated clearance of amyloid deposition in an Alzheimer disease model^47^; to control tumour growth by reactivating myeloid cells in glioblastoma,^48^ and to enhance microglia-mediated clearance in models of CNS demyelination.^49^ More recently, niacin has gained even more attention as a nicotinamide adenine dinucleotide (NAD^+^) boosting molecule, similarly to NMN (Nicotinamide mononucleotide) and NR (Nicotinamide riboside) , to delay the disease effects in models of different neurodegenerative disorders including Alzheimer and Parkinson.^50^ More importantly, a first study conducted in humans, recently suggested that niacin significantly ameliorates the disease-specific hallmarks in patients with adult-onset mitochondrial myopathy, among which increased NAD^+^ levels in blood and skeletal muscle.^51^ These reported effects are in line with the reduction of NCV and fibre/axonal degeneration that we observed following Niacin-SR long-term administration in *Mtmr2* KO mice. If this is a result of improvement of mitochondrial associated functions and NAD+ availability in treated nerves remains to be assessed.

## Conclusion

In conclusion, our results suggest that, using the nerve crush injury as experimental setting and the *Mtmr2* KO mouse mutant as a model, niacin treatment is not detrimental to nerve regeneration. Unexpectedly, the *Mtmr2* KO mouse revealed a defect in nerve regeneration which was not previously reported. Moreover, by treating *Mtmr2* KO mice for 5 months using an extended-release formulation of niacin, Niacin-SR, we found that the treatment increases NCV and slightly reduces the number of degenerating fibres, with a significant inverse correlation between NCV and plasma NF-L levels. However, the number of fibres carrying aberrant myelin did not change following treatment. This limited efficacy might be due to the drug formulation and to the model, the *Mtmr2* KO mouse that does not fully recapitulate human CMT4B1 characterized by a very severe phenotype with infantile onset. Further experiments using different disease models are necessary to assess whether Niacin-SR may preserve nerve fibres from degeneration.

## Supplementary Material

fcaf039_Supplementary_Data

## Data Availability

RNA-seq data have been deposited in NCBI (GSE233289).
