# Supplementary material for: Niacin ameliorates Charcot-Marie-Tooth 4B1 neuropathy without interfering with nerve regeneration
Source: Brain Commun. 2025 Jan 31;7(1):fcaf039. doi: 10.1093/braincomms/fcaf039 (PMC11803425; doi:10.1093/braincomms/fcaf039)
Supplement: fcaf039_Supplementary_Data [file fcaf039_supplementary_data.zip › Legends_for_Supplementary_Material_2_and_3.docx]

**Supplementary_material_2. Gene Set Enrichment Analysis of *Mtmr2* KO sciatic nerve RNA-seq analysis.** GSEA analysis of the RNA-seq analysis is shown for gene sets that are preferentially increased in wild type mice vs. gene sets that are elevated in *Mtmr2* KO sciatic nerve.

**Supplementary_material_3. Gene Ontology Analysis of Genes decreased in *Mtmr2* KO sciatic nerve.** The table shows the complete gene ontology analysis of downregulated genes as performed by Enrichr, as described in the Methods section, including a list of genes within enriched categories.
