# Supplementary material for: Niacin ameliorates Charcot-Marie-Tooth 4B1 neuropathy without interfering with nerve regeneration
Source: Brain Commun. 2025 Jan 31;7(1):fcaf039. doi: 10.1093/braincomms/fcaf039 (PMC11803425; doi:10.1093/braincomms/fcaf039)

Supplementary_material_1

Supplementary Figure 1. TACE activity in intact and crushed sciatic nerves from control mice treated with niacin.

(A) TACE (TACE, TNF-Alpha Convertase Enzyme) activity from intact sciatic nerve lysates following pure niacin administration by intraperitoneal (i.p.) injection at 120 mg/Kg, one-tailed Mann Whitney t-test, p=0.0385, n=9 animals per genotype. One-tailed test was applied assuming niacin is known to increase TACE activity. (B) TACE activity from crushed sciatic nerve lysates following pure niacin administration by i.p. injection at 120 mg/Kg, two-tailed Mann Whitney t-test, p=0.274, n=5 animals per genotype. Data are mean ± SEM. RFU is relative fluorescence unit.

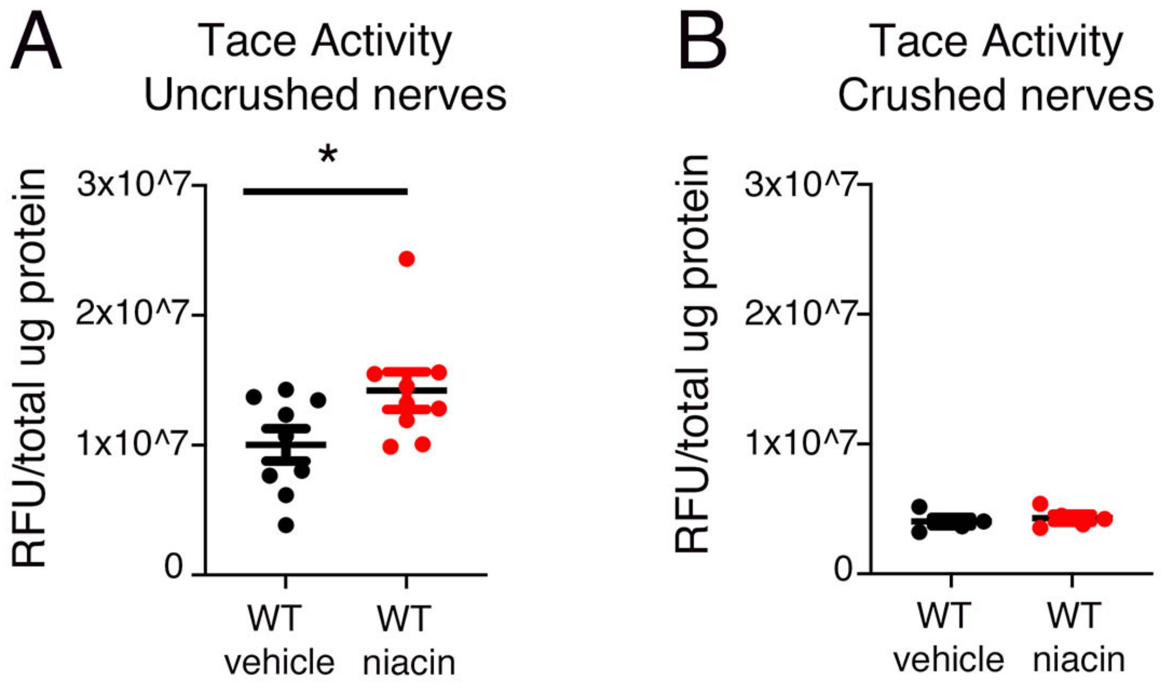

Supplementary Figure 2. Autophagic markers in *Mtmr2* KO crushed sciatic nerves. (A) Western blot analysis of sciatic nerves lysates from WT and KO at 3dpi, distal stump, to measure LC3II/I and NBR1 expression levels as quantified in (A'): LC3II/I expression in mutant nerves as compared to wildtype set to 1, two-tailed one sample t-test, n=5 animals analyzed per genotype in two different experiments; p=0.211, t=1.487, df=4; NBR1 expression in mutant nerves as compared to wildtype set to 1, two-tailed one sample t-test, n=5 animals analyzed per genotype in two different experiments; p=0.422, t=0.894, df=4.

(B) Western blot analysis of sciatic nerves lysates from WT and KO at 7dpi, distal stump, to measure LC3II/I and NBR1 expression levels loaded on the same gel, as quantified in (B'): LC3II/I, two-tailed Mann Whitney t-test, p>0.999, n=3 animals per genotype; NBR1, two-tailed Mann Whitney t-test, p>0.999, n=3 animals per genotype. Raw data are displayed in Supplementary Figure 7 and Supplementary Figure 8. Data are mean ± SEM.

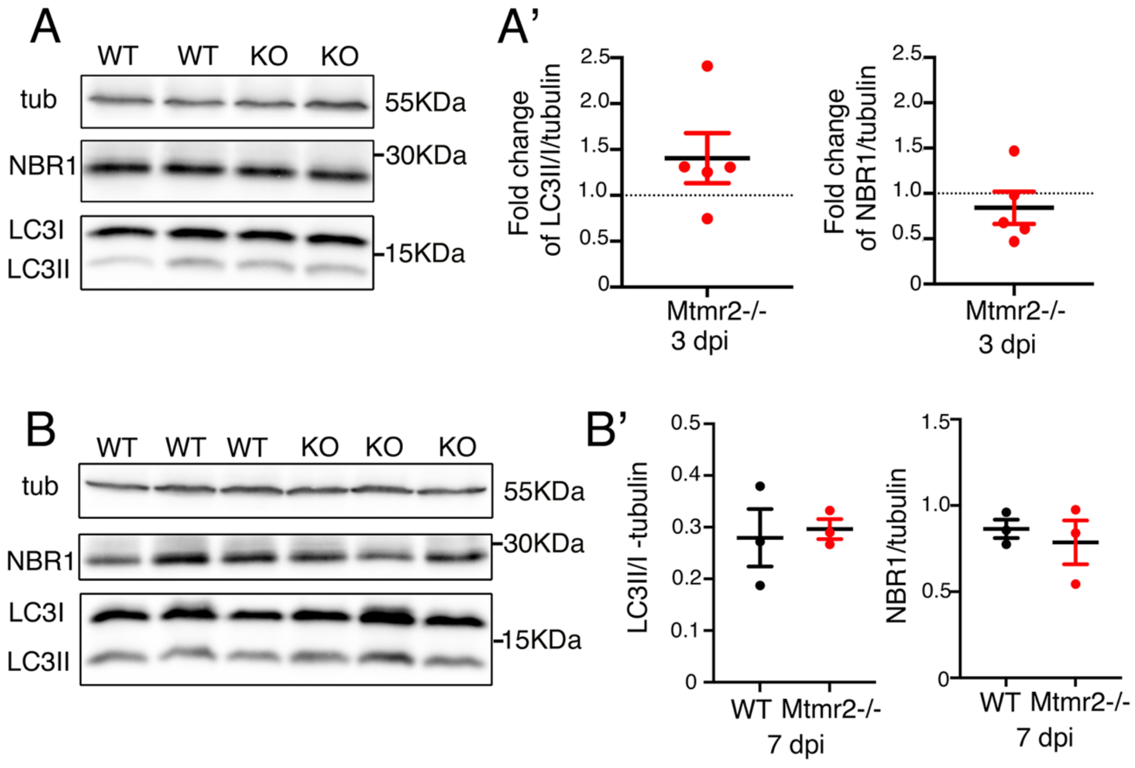

Supplementary Figure 3. Heat map of Gene Expression Profiles. The heat map indicates the relative expression for each nerve sample (n=4) within the 3 groups: control, *Mtmr2* KO, and niacin-treated *Mtmr2* KO sciatic nerves. The heat map includes all genes.

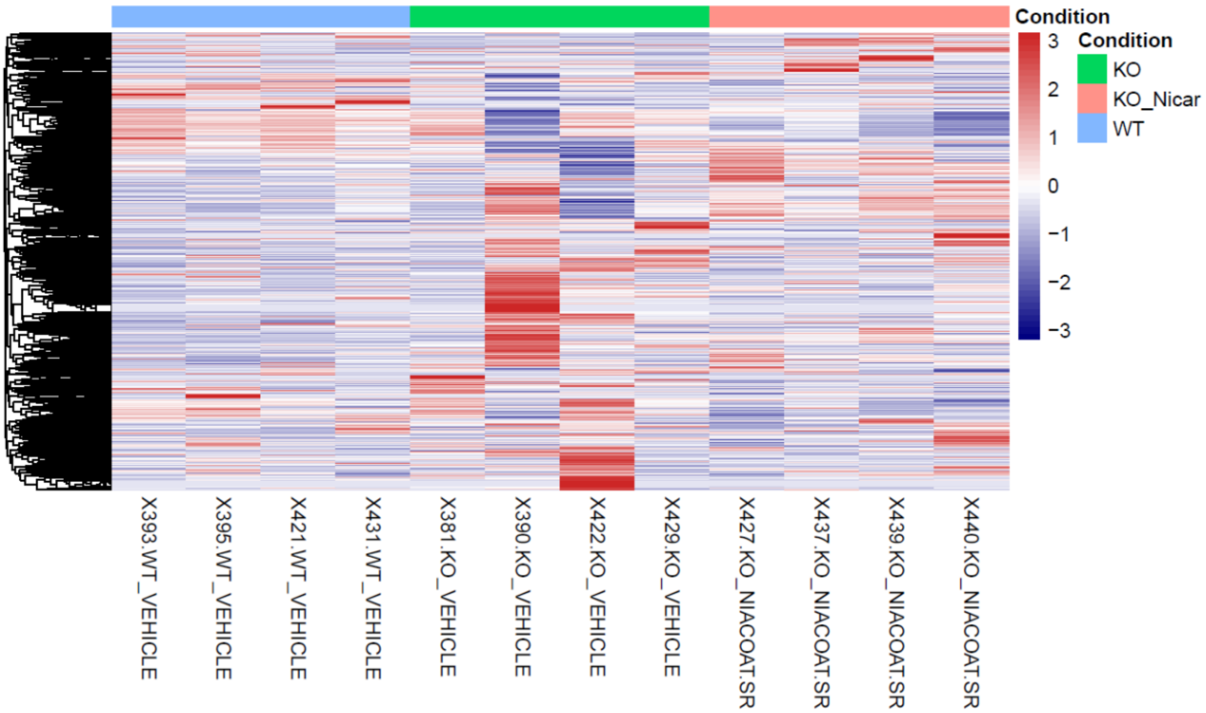

Supplementary Figure 4. Biochemical analysis from blood of vehicle or Niacin-SR treated mice at 6 months. Non-parametric one-way Anova, Kruskal-Wallis test, Dunn's multiple comparison test, n= 5 mice per genotype/condition of treatment, p=0.131 (A); p=0.0541 (B), p=0.073 (C), p=0.438 (D), p=0.385 (E), p=0.151 (F), p=0.346 (G) and p=0.04 (H). Comparisons of pairs within the four groups are statistically not significant. ALT, Alanine transaminase; AST, Aspartate transaminase; HDL, High density lipoprotein; LDL, Low density lipoprotein. Data are mean ± SEM.

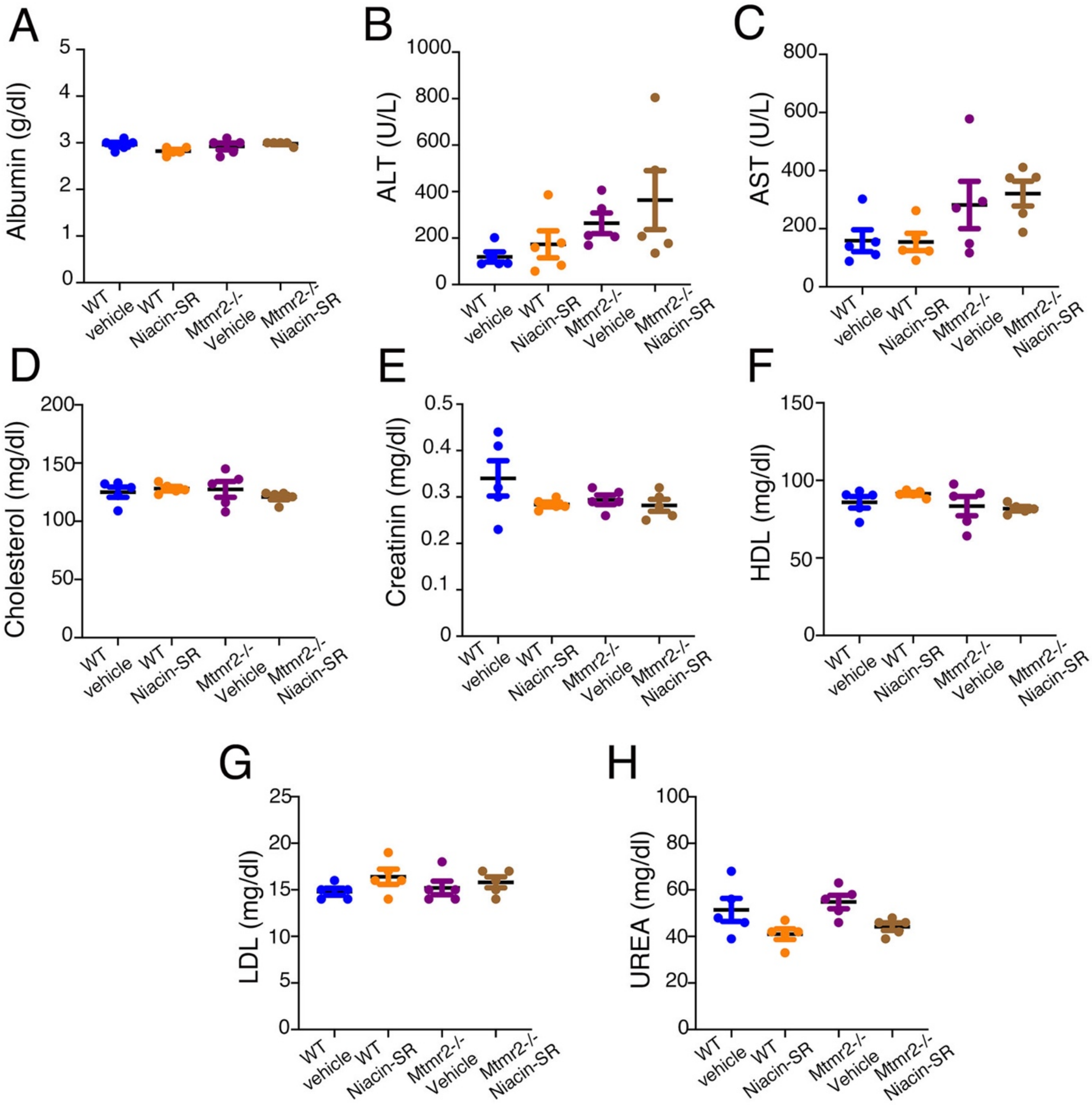

Supplementary Figure 5. Histological analysis of kidney, liver and gut following long-term treatment with Niacin-SR. No gross histological alterations have been observed in tissues from 6-months old WT and *Mtmr2* KO treated using Niacin-SR. Bar is 49 μm for kidney and liver and 98 μm for gut images. Niacin-SR is slow release.

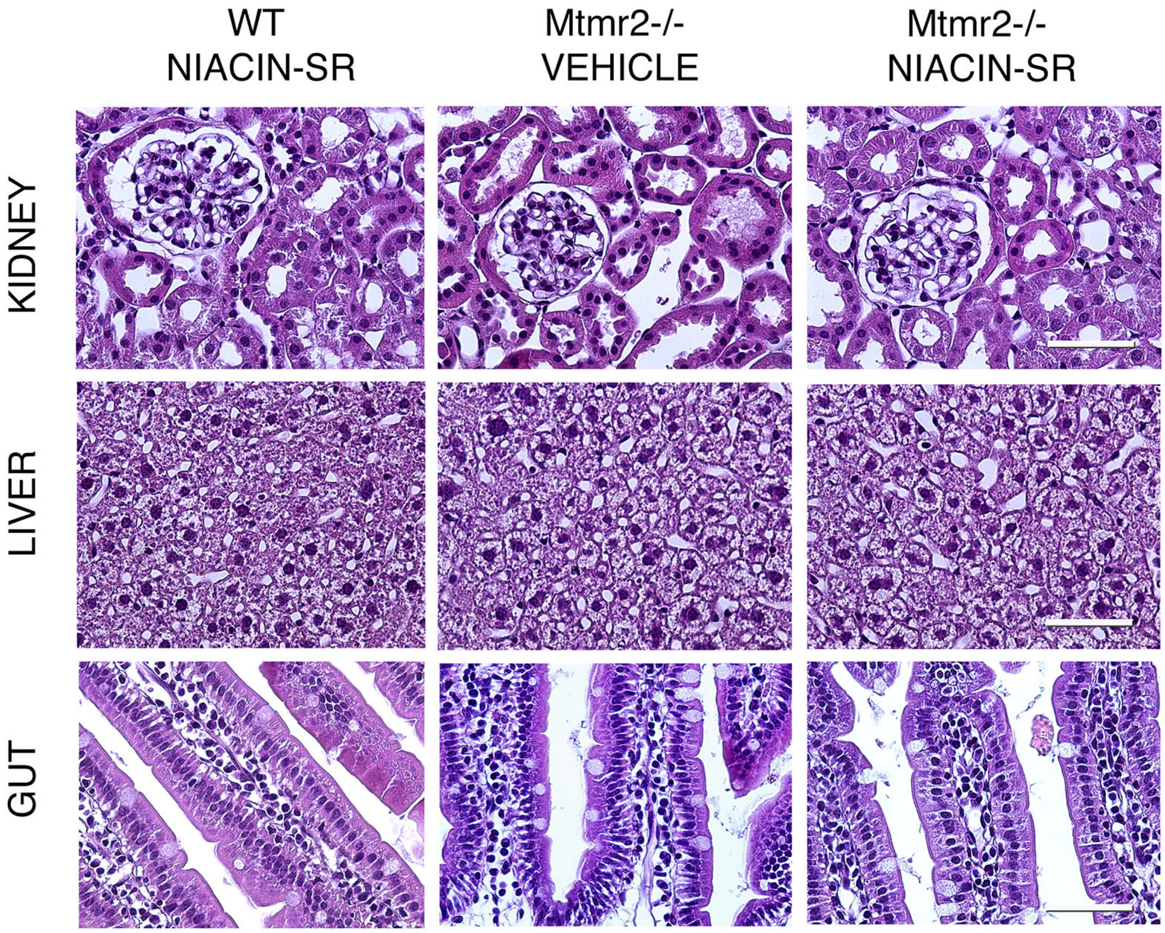

Supplementary Figure 6. Sciatic nerve semithin sections at high magnification showing examples of aberrant and degenerating fibers in Mtmr2 KO mice at P180. Aberrant fibers, (myelin outfoldings, infoldings and tomacula) were identified as following: myelin outfoldings (pink asterisks) appear as satellite axons around the main myelinated axon, with whom they share the same myelin thickness and basal lamina. Myelin infoldings (orange asterisk) are similar structures which instead develop towards the axon. Tomacula (green asterisks) appear as focally myelin thickenings along single nerve fibers which often show first signs of degeneration. Degenerating fibers (blue-azure asterisks) include fibers showing axonal shrinkage and displacement (e.g fibers with degenerating tomacula), accumulation of internal myelin debris and vacuolar-like structures.

A

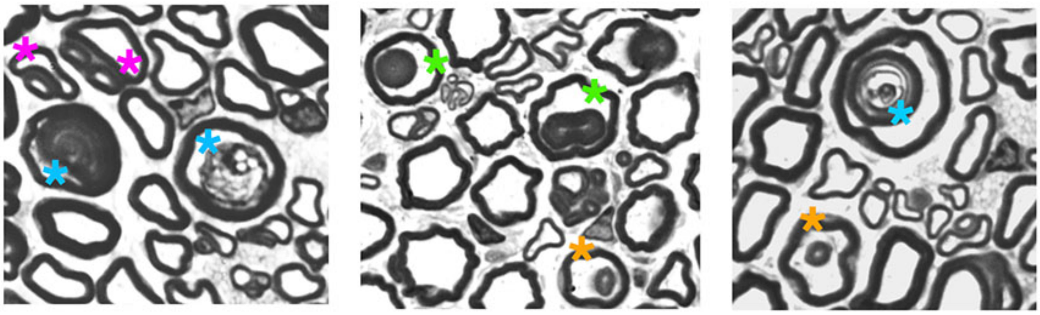

Supplementary Figure 7. Uncropped images of western blot experiments displayed in Supplementary Figure 2, panel A.

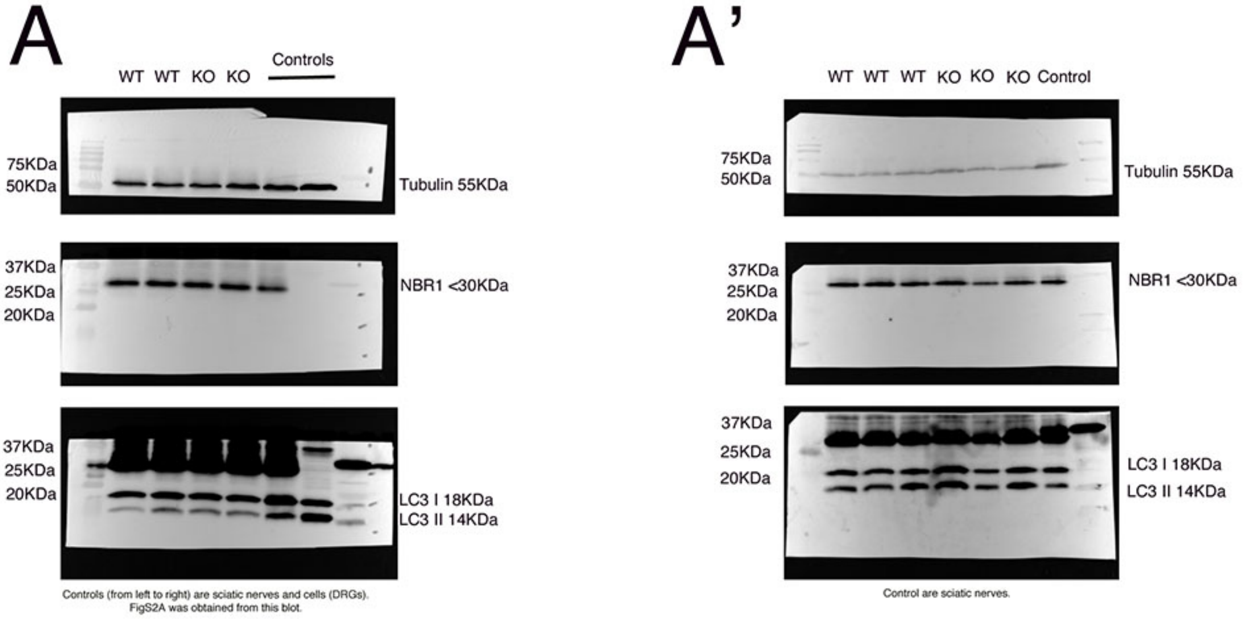

Supplementary Figure 8. Uncropped images of western blot experiments displayed in Supplementary Figure 2, panel B.

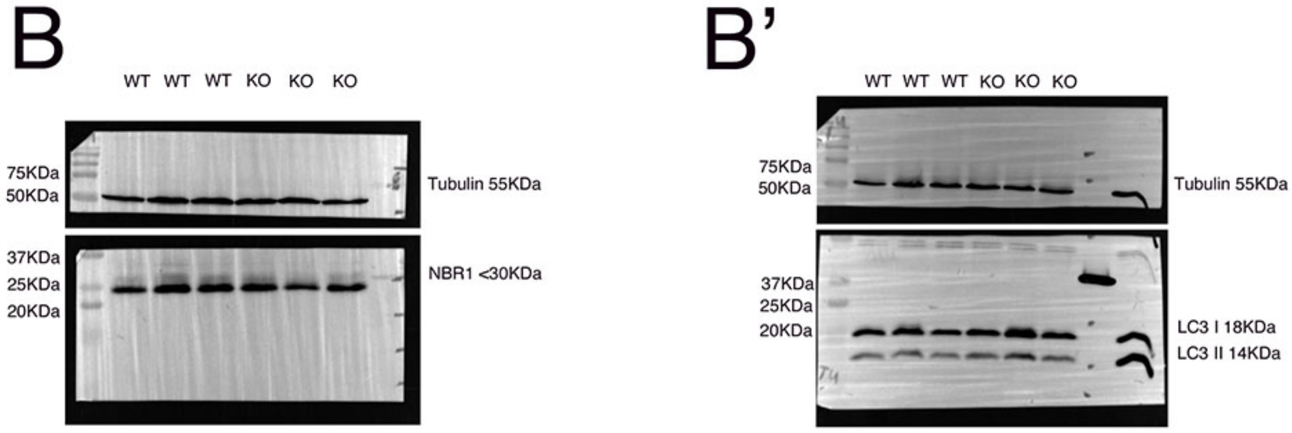

Supplement: fcaf039_Supplementary_Data [file fcaf039_supplementary_data.zip › Supplementary_material_1.pdf]
