# Supplementary material for: Niacin ameliorates Charcot-Marie-Tooth 4B1 neuropathy without interfering with nerve regeneration
Source: Brain Commun. 2025 Jan 31;7(1):fcaf039. doi: 10.1093/braincomms/fcaf039 (PMC11803425; doi:10.1093/braincomms/fcaf039)
Supplement: fcaf039_Supplementary_Data [file fcaf039_supplementary_data.zip › Supplementary_material_2.pdf]

Table: Genes Enriched in WT

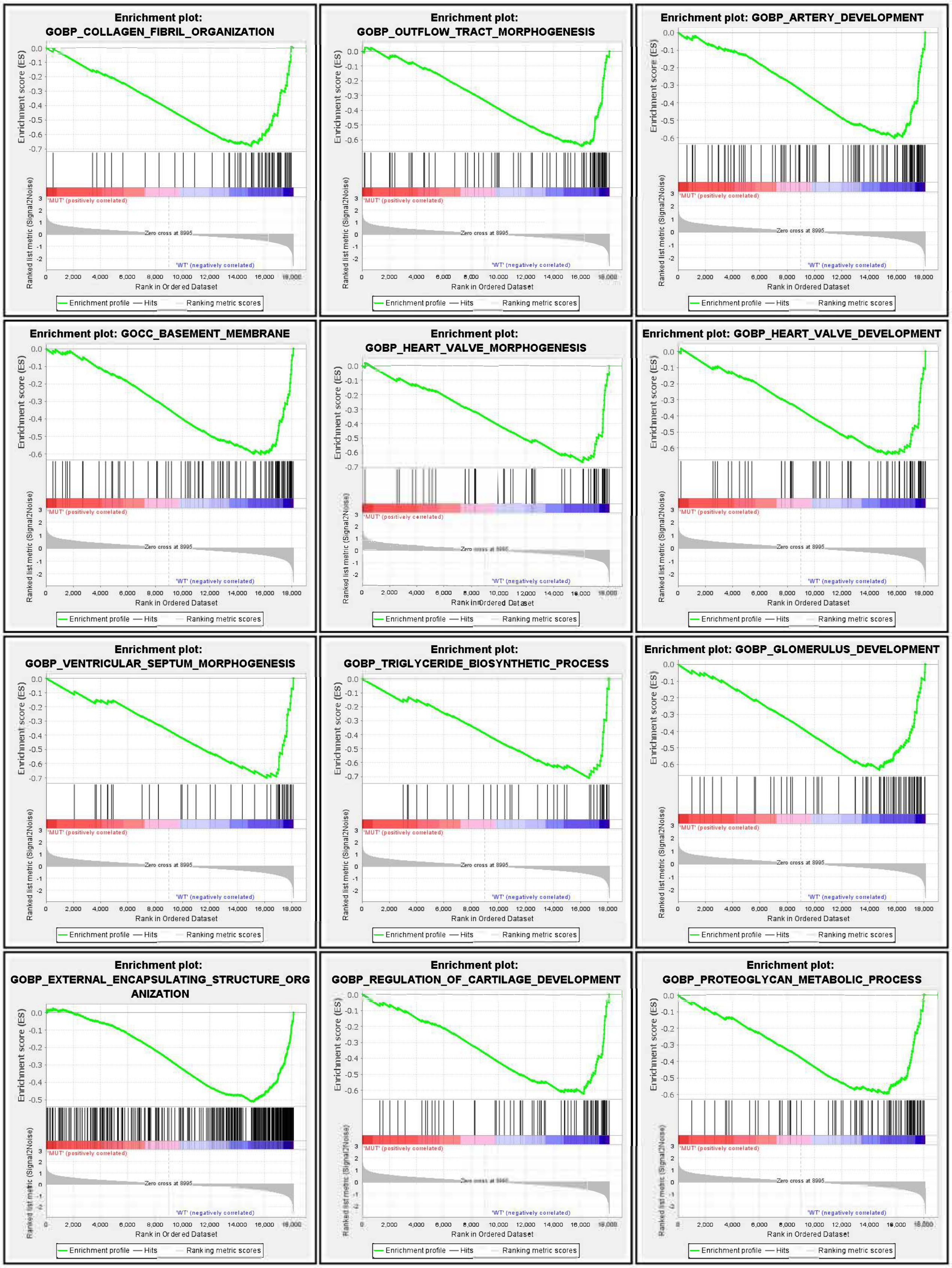

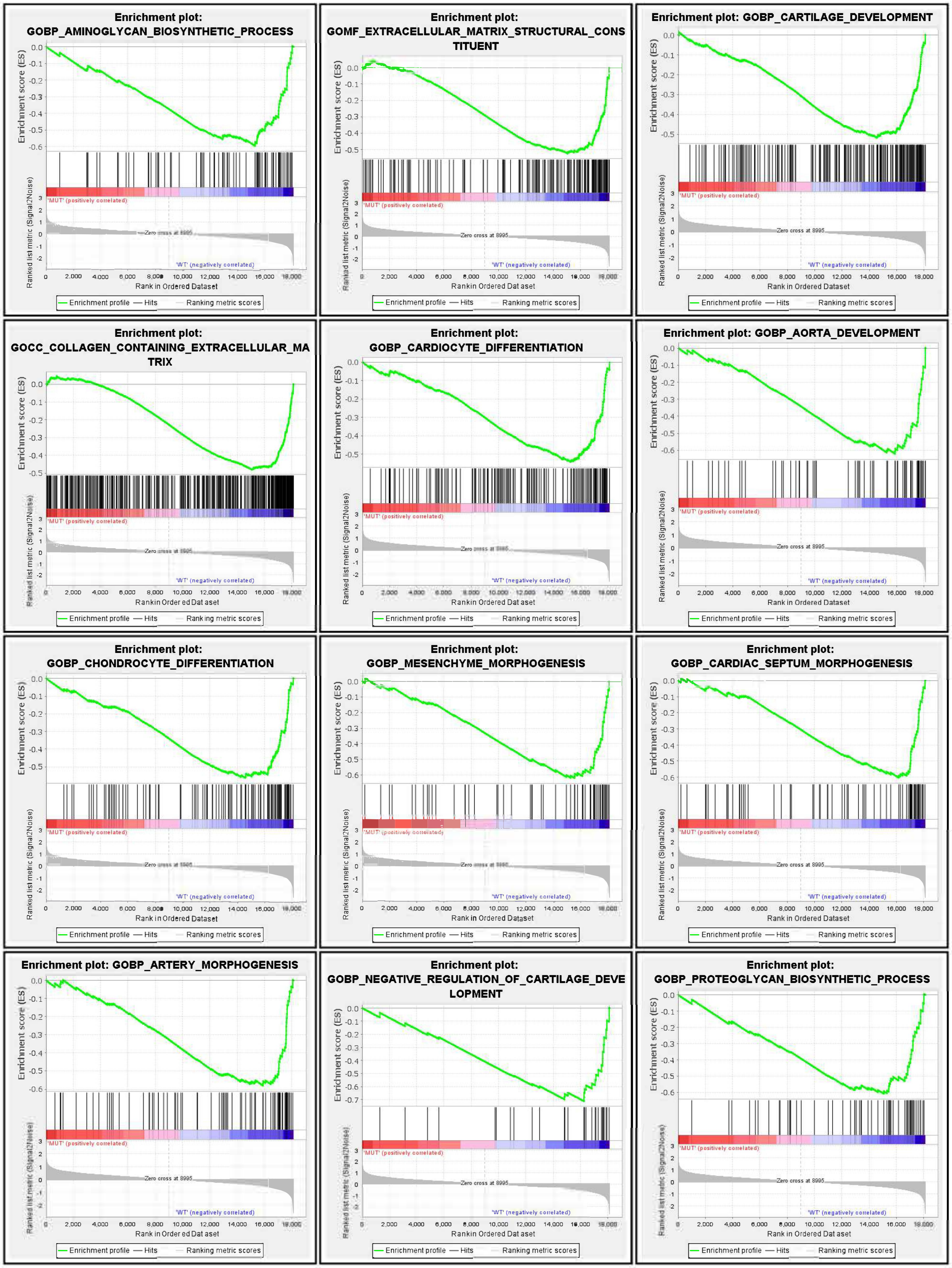

Enrichment plot: GOBP_AMINOGLYCAN_BIOSYNTHETIC_PROCESS
Enrichment plot: GOMF_EXTRACELLULAR_MATRIX_STRUCTURAL_CONSTITUENT
Enrichment plot: GOBP_CARTILAGE_DEVELOPMENT
Enrichment plot: GOCC_COLLAGEN_CONTAINING_EXTRACELLULAR_MATRIX
Enrichment plot: GOBP_CARDIOCYTE_DIFFERENTIATION
Enrichment plot: GOBP_AORTA_DEVELOPMENT
Enrichment plot: GOBP_CHONDROCYTE_DIFFERENTIATION
Enrichment plot: GOBP_MESENCHYME_MORPHOGENESIS
Enrichment plot: GOBP_CARDIAC_SEPTUM_MORPHOGENESIS
Enrichment plot: GOBP_ARTERY_MORPHOGENESIS
Enrichment plot: GOBP_NEGATIVE_REGULATION_OF_CARTILAGE_DEVELOPMENT
Enrichment plot: GOBP_PROTEOGLYCAN_BIOSYNTHETIC_PROCESS
Enrichment score (ES)
Ranked list metric (Signal2Noise)
'MUT' (positively correlated)
Zero cross at 8995
'WT' (negatively correlated)
Rank in Ordered Dataset
Enrichment profile
Hits
Ranking metric scores

GSEA downregulated genes:

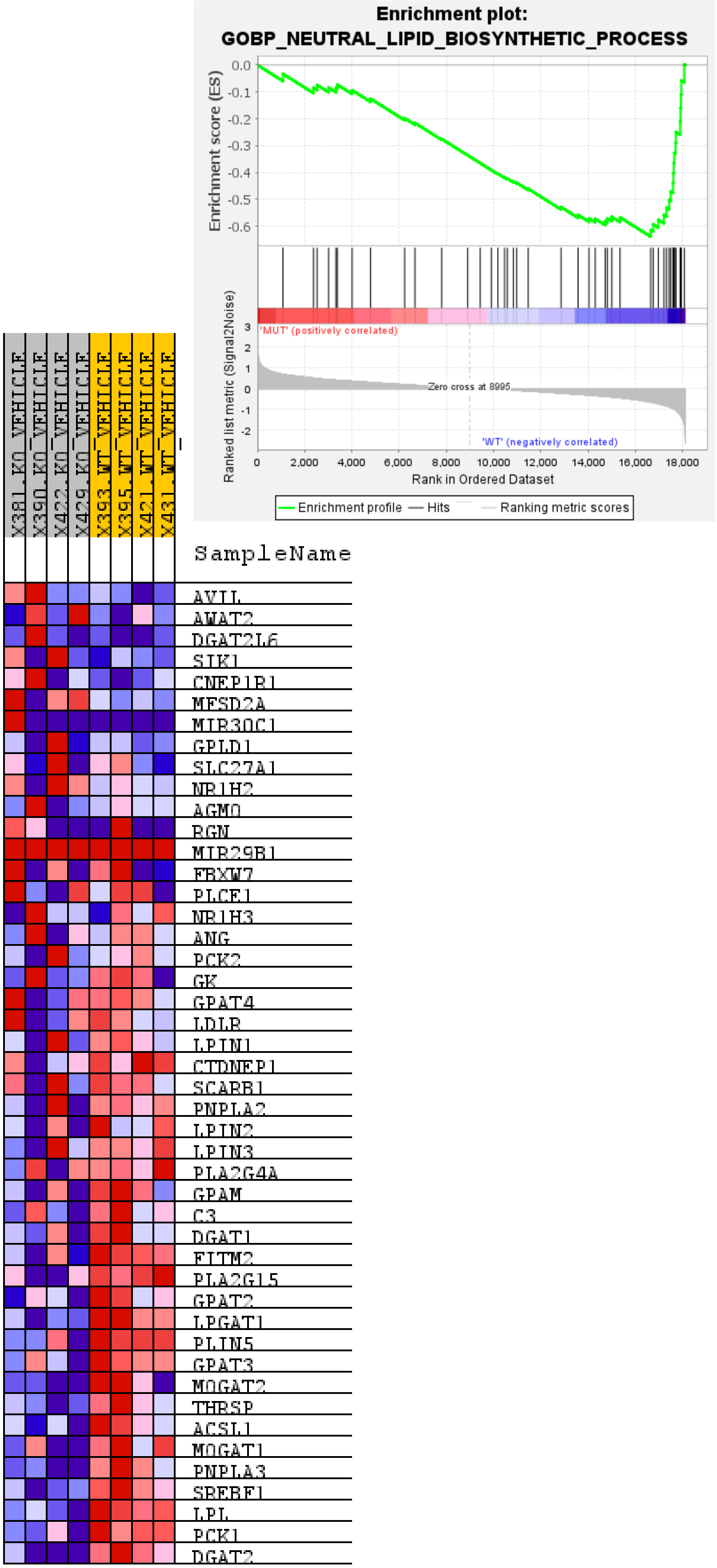

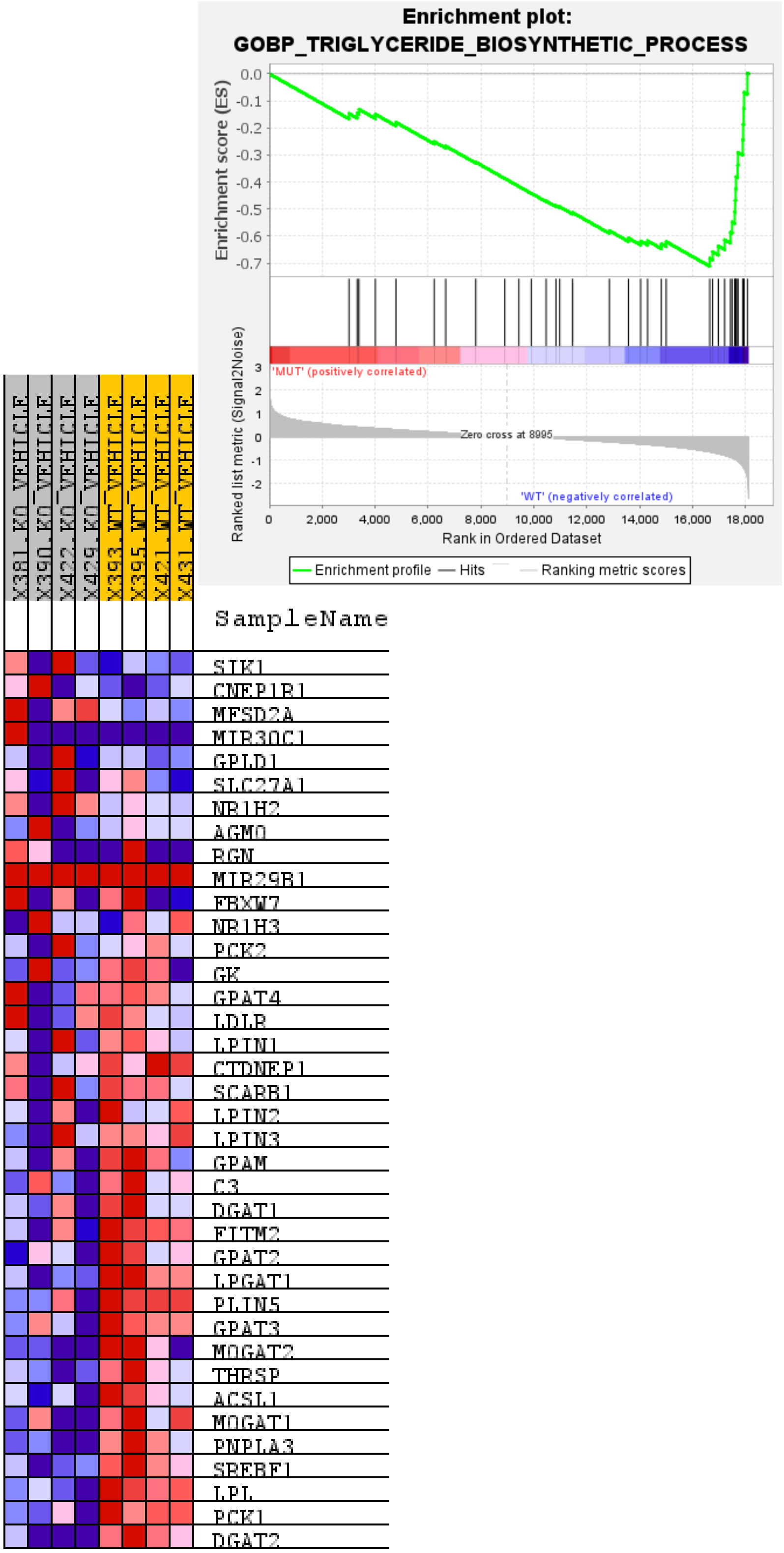

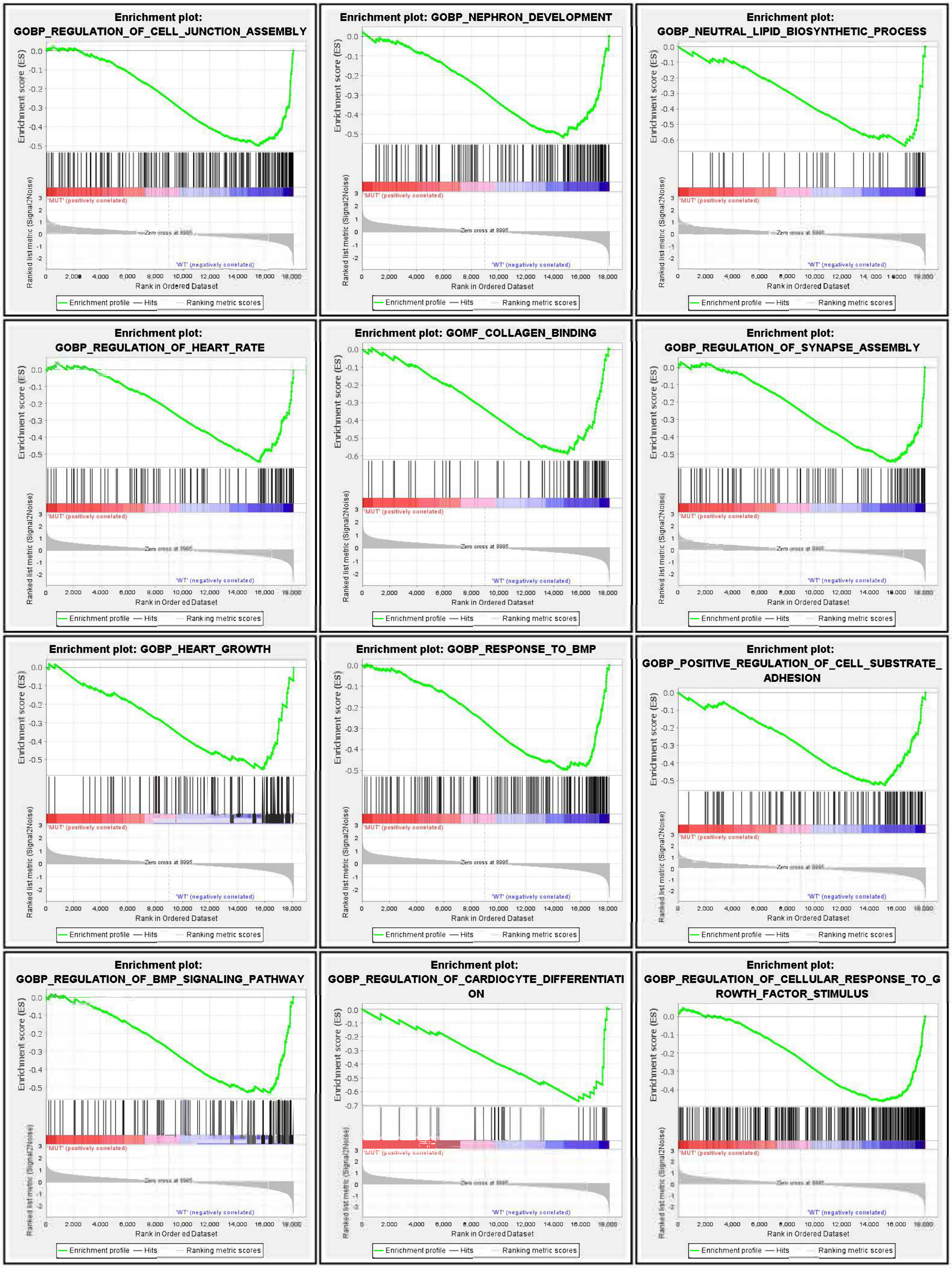
Enrichment plot: GOBP_REGULATION_OF_CELL_JUNCTION_ASSEMBLY
Enrichment plot: GOBP_NEPHRON_DEVELOPMENT
Enrichment plot: GOBP_NEUTRAL_LIPID_BIOSYNTHETIC_PROCESS
Enrichment plot: GOBP_REGULATION_OF_HEART_RATE
Enrichment plot: GOMF_COLLAGEN_BINDING
Enrichment plot: GOBP_REGULATION_OF_SYNAPSE_ASSEMBLY
Enrichment plot: GOBP_HEART_GROWTH
Enrichment plot: GOBP_RESPONSE_TO_BMP
Enrichment plot: GOBP_POSITIVE_REGULATION_OF_CELL_SUBSTRATE_ADHESION
Enrichment plot: GOBP_REGULATION_OF_BMP_SIGNALING_PATHWAY
Enrichment plot: GOBP_REGULATION_OF_CARDIOCYTE_DIFFERENTIATION
Enrichment plot: GOBP_REGULATION_OF_CELLULAR_RESPONSE_TO_GROWTH_FACTOR_STIMULUS
Enrichment score (ES)
Ranked list metric (Signal2Noise)
'MUT' (positively correlated)
'WT' (negatively correlated)
Zero cross at 8995
Rank in Ordered Dataset
Enrichment profile
Hits
Ranking metric scores

Table: Genes enriched in Mtmr2 -/-

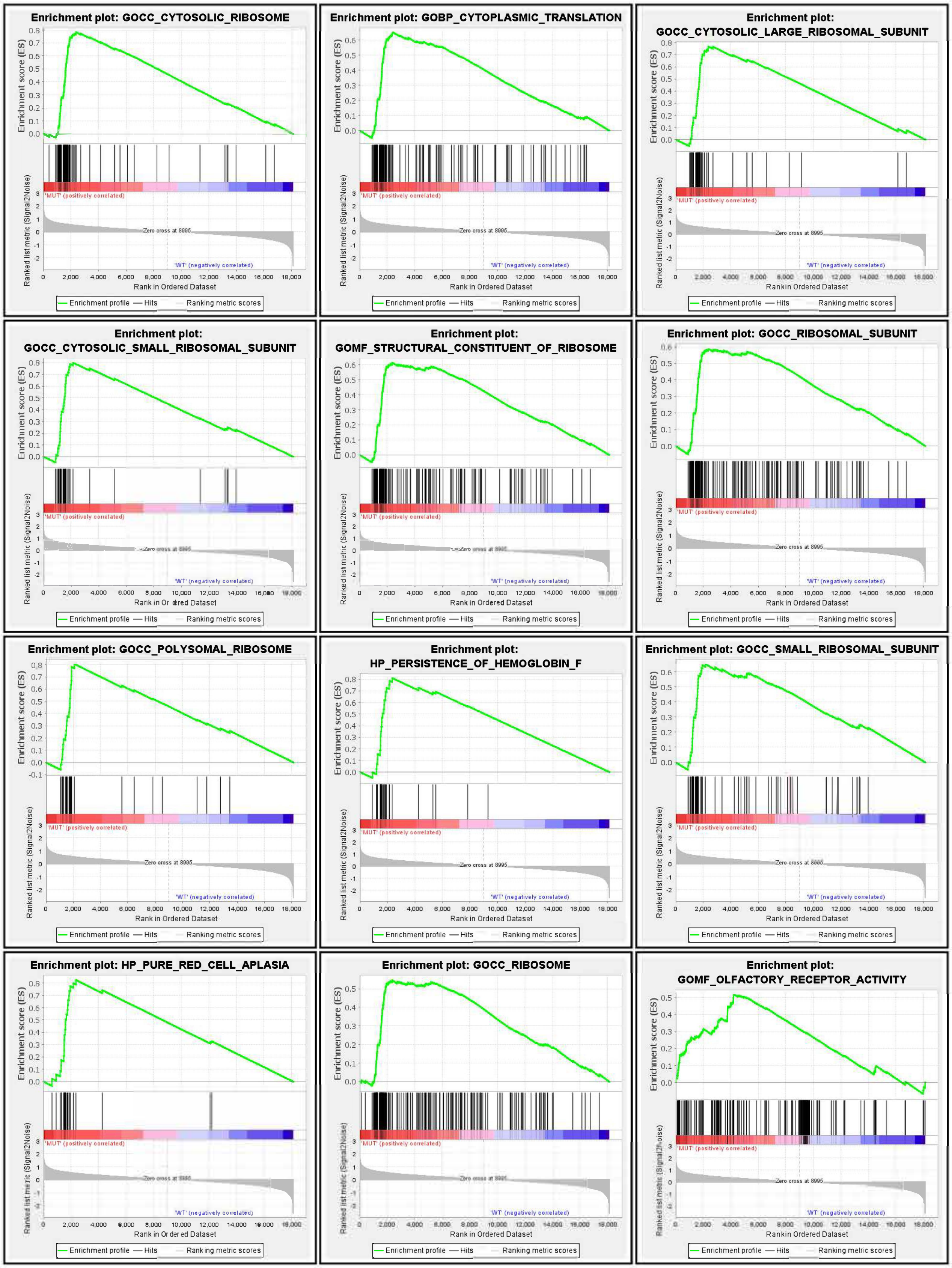

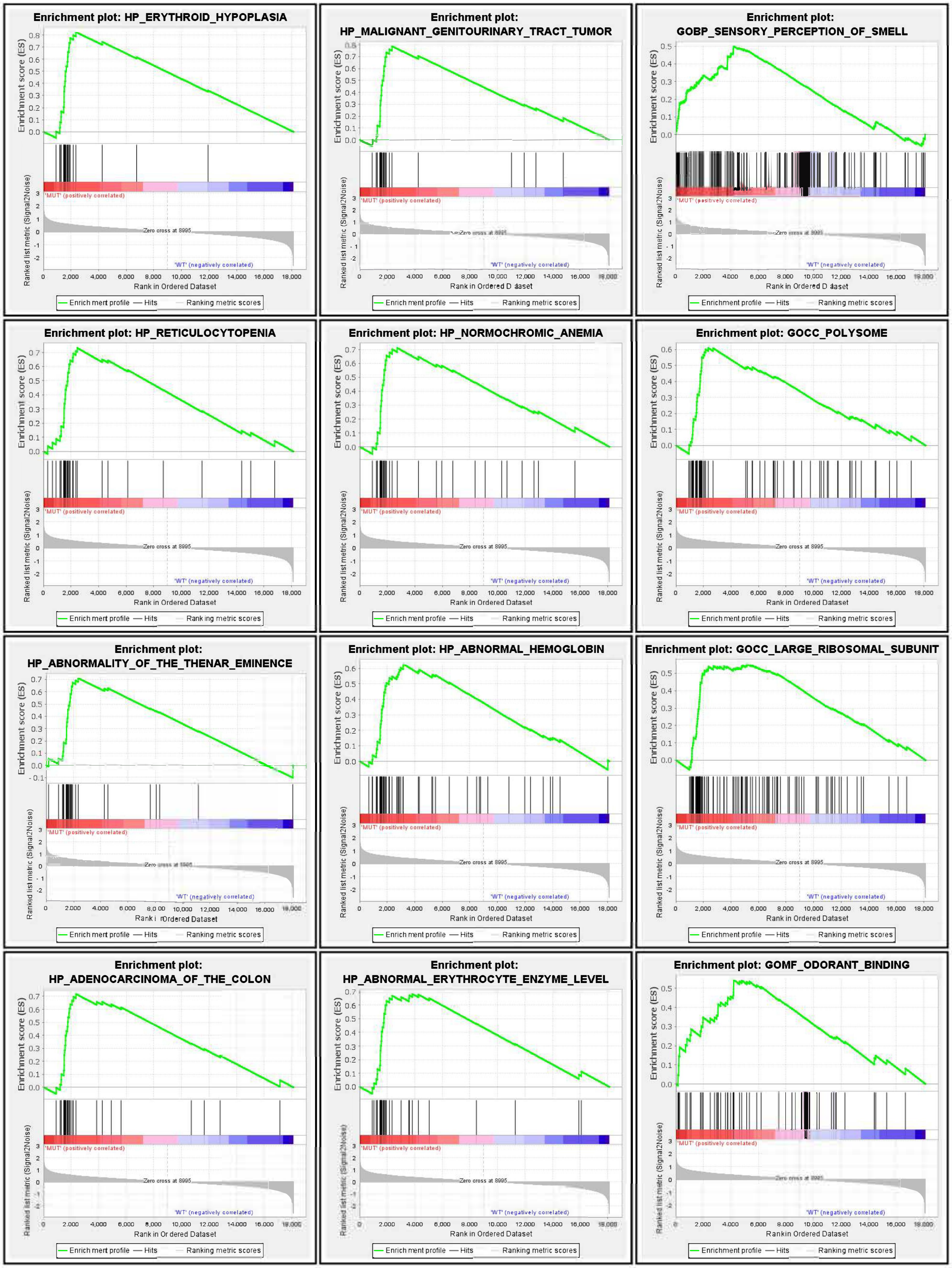
Enrichment plot: HP_ERYTHROID_HYPOPLASIA
Enrichment plot: HP_MALIGNANT_GENITOURINARY_TRACT_TUMOR
Enrichment plot: GOBP_SENSORY_PERCEPTION_OF_SMELL
Enrichment plot: HP_RETICULOCYTOPENIA
Enrichment plot: HP_NORMOCHROMIC_ANEMIA
Enrichment plot: GOCC_POLYSOME
Enrichment plot: HP_ABNORMALITY_OF_THE_THENAR_EMINENCE
Enrichment plot: HP_ABNORMAL_HEMOGLOBIN
Enrichment plot: GOCC_LARGE_RIBOSOMAL_SUBUNIT
Enrichment plot: HP_ADENOCARCINOMA_OF_THE_COLON
Enrichment plot: HP_ABNORMAL_ERYTHROCYTE_ENZYME_LEVEL
Enrichment plot: GOMF_ODORANT_BINDING
Enrichment score (ES)
Ranked list metric (Signal2Noise)
'MUT' (positively correlated)
'WT' (negatively correlated)
Zero cross at 8995
Rank in Ordered Dataset
Enrichment profile
Hits
Ranking metric scores
