# Supplementary material for: Niacin ameliorates Charcot-Marie-Tooth 4B1 neuropathy without interfering with nerve regeneration
Source: Brain Commun. 2025 Jan 31;7(1):fcaf039. doi: 10.1093/braincomms/fcaf039 (PMC11803425; doi:10.1093/braincomms/fcaf039)
Supplement: fcaf039_Supplementary_Data [file fcaf039_supplementary_data.zip › Supplementary_material_3.pdf]

Term	Overlap	P-value	Adjusted P-value	Odds Ratio	Combined Score	Genes
Adipogenesis WP236	15/131	1,03E-09	3,45E-07	9,013989	186,54627	GDF10;SREBF1;CEBPA;UCP1;LPL;TWIST1;AGPAT2;BMP4;LIPE;BMP1;LEP;PNPLA3;PPARG;PLIN1;PCK1
Familial Partial Lipodystrophy WP5102	7/30	2,20E-07	3,69E-05	20,72619	317,69667	CEBPA;LIPE;SREBF1;PRRX1;LPL;PPARG;PLIN1
Differentiation Of White And Brown Adipocyte WP2895	6/25	1,40E-06	1,56E-04	21,43557	288,99107	BMP4;CEBPA;LEP;EBF3;PPARG;SLC7A10
PI3K Akt Signaling Pathway WP4172	18/338	3,22E-06	2,70E-04	3,922122	49,599146	NTRK2;FLT4;PPP2R5A;EFNA5;THBS2;THBS1;CCND2;PPP2R1B;CREB3L1;NTF3;COL4A6;ITGA8;COL4A5;COL6A6;COL6A5;TEK;PCK1;FGF10
Triacylglyceride Synthesis WP325	5/24	2,32E-05	1,55E-03	17,80159	189,99609	LIPE;DGAT2;GPD1;LPL;AGPAT2
Thermogenesis WP4321	9/108	3,28E-05	1,83E-03	6,21001	64,122437	LIPE;NPR1;CNR1;CREB3L1;UCP1;ADCY3;PPARG;PLIN1;ADCY5
Major Receptors Targeted By Epinephrine And Norepinephrine WP4589	4/16	7,44E-05	3,56E-03	22,47945	213,70482	ADCY3;ADRA1D;ADRA2A;ADCY5
miR 509 3P Alteration Of YAP1 ECM Axis WP3967	4/17	9,61E-05	4,02E-03	20,74921	191,93214	EDNRA;TWIST1;THBS2;GPC6
Focal Adhesion PI3K Akt mTOR Signaling Pathway WP3932	14/302	1,76E-04	6,53E-03	3,346927	28,942595	SREBF1;PFKFB3;FLT4;PPP2R5A;EFNA5;THBS2;THBS1;LIPE;PPP2R1B;CREB3L1;COL4A6;ITGA8;TEK;FGF10
Development Of Ureteric Collection System WP5053	6/60	2,56E-04	8,49E-03	7,528736	62,274966	BMP4;MYCN;FRAS1;FST;ITGA8;ROBO1
Transcription Factor Regulation In Adipogenesis WP3599	4/22	2,79E-04	8,49E-03	14,98174	122,63458	CEBPA;LEP;TWIST1;PPARG
Thyroid Hormones Production And Peripheral Downstream Signaling Effects WP4746	7/93	4,63E-04	1,20E-02	5,525308	42,418525	NPR1;UCP1;AQP7;ADCY3;PPARG;SLC16A10;PLIN1
PPAR Signaling Pathway WP3942	6/67	4,67E-04	1,20E-02	6,662408	51,088558	UCP1;AQP7;LPL;PPARG;PLIN1;PCK1
Transcriptional Cascade Regulating Adipogenesis WP4211	3/13	8,22E-04	0,019673	20,16451	143,24014	CEBPA;SREBF1;PPARG
Cell Migration And Invasion Through p75NTR WP4561	4/30	9,51E-04	0,021242	10,36776	72,137416	NTRK2;TWIST1;EFNA5;MMP9
Monoamine GPCRs WP58	4/33	0,001372	0,028718	9,293812	61,262682	CHRM3;HTR7;ADRA1D;ADRA2A
GPCRs Class A Rhodopsin Like WP455	11/259	0,001731	0,034109	3,027957	19,255114	CHRM3;OXTR;EDNRA;HTR7;HCAR1;CNR1;PTGER3;APLNR;ADRA1D;ADRA2A;CMKLR1
GPCRs Other WP117	6/91	0,002325	0,04327	4,775416	28,958405	CHRM3;EDNRA;HTR7;CNR1;ADRA1D;ADGRL2
Nuclear Receptors Meta Pathway WP2882	12/314	0,002621	0,046204	2,714579	16,136486	SLC5A6;SLC6A6;SREBF1;THBD;SRPX2;ALAS1;SLC2A13;MYOF;AMIGO2;PCK1;ESR1;CAP2
